# The Neurofunctional Correlates of Morphosyntactic and Thematic Impairments in Aphasia: A Systematic Review and Meta-analysis

**DOI:** 10.1007/s11065-024-09648-0

**Published:** 2024-08-31

**Authors:** Sabrina Beber, Giorgia Bontempi, Gabriele Miceli, Marco Tettamanti

**Affiliations:** 1 Center for Mind/Brain Sciences (CIMeC), University of Trento, Rovereto, TN 38122 Italy; 2 University of Milano-Bicocca, Milano, MI 20126 Italy

**Keywords:** Morphosyntactic processing, Thematic role assignment, Sentence processing, Neuropsychological assessment of language abilities, Lesion-symptom mapping, Aphasia

## Abstract

Lesion-symptom studies in persons with aphasia showed that left temporoparietal damage, but surprisingly not prefrontal damage, correlates with impaired ability to process thematic roles in the comprehension of semantically reversible sentences (*The child is hugged by the mother*). This result has led to challenge the time-honored view that left prefrontal regions are critical for sentence comprehension. However, most studies focused on thematic role assignment and failed to consider morphosyntactic processes that are also critical for sentence processing. We reviewed and meta-analyzed lesion-symptom studies on the neurofunctional correlates of thematic role assignment and morphosyntactic processing in comprehension and production in persons with aphasia. Following the PRISMA checklist, we selected 43 papers for the review and 27 for the meta-analysis, identifying a set of potential bias risks. Both the review and the meta-analysis confirmed the correlation between thematic role processing and temporoparietal regions but also clearly showed the involvement of prefrontal regions in sentence processing. Exploratory meta-analyses suggested that both thematic role and morphosyntactic processing correlate with left prefrontal and temporoparietal regions, that morphosyntactic processing correlates with prefrontal structures more than with temporoparietal regions, and that thematic role assignment displays the opposite trend. We discuss current limitations in the literature and propose a set of recommendations for clarifying unresolved issues.

**Supplementary Information:**

The online version contains supplementary material available at 10.1007/s11065-024-09648-0.

## Introduction

Sentence processing disorders are a common occurrence in aphasia. Throughout the years, they have given cognitive neuroscientists the opportunity to query the functional architecture of the language system and to investigate its neural correlates.

Until the second half of the twentieth century, research studies on sentence-processing disorders focused on agrammatic speech (e.g., Goodglass et al., 1972; Tissot et al., 1973). These studies either neglected to study comprehension or implicitly took it to be completely independent from production (Lenneberg, 1973; Locke et al., 1973). This approach was challenged by papers showing that aphasic disorders also affect sentence comprehension and that grammatical difficulties in comprehension and production often (but, not always) co-occur in the same individual (Caramazza & Zurif, 1976). Two critical aspects of sentence processing, involved in both comprehension and production, have attracted attention, namely the assignment of thematic roles (e.g., theme, agent; see section “The Assignment of Thematic Roles”) and the processing of morphosyntax (e.g., free-standing and grammatical morphemes; see section “Morphosyntactic Processes”).

### The Assignment of Thematic Roles

Thematic role assignment is necessary to establish *who does what to whom*, that is, to identify the agent (the doer) and the theme (the recipient) of the action. This goal is easily achieved in semantically irreversible sentences, in which agent and theme can be assigned by encyclopedic knowledge, even if syntactic analysis is disrupted. For example, given the act of eating, a boy and an apple, only a sentence like *The boy is eating an apple* or *The apple is eaten by the boy* makes common sense. Thematic role assignment is more demanding in the case of semantically reversible sentences, in which more than one noun can be the doer or the receiver of the action. For example, given a man, a woman and the act of hugging, both *The man is hugging the woman* and *The woman is hugging the man* are plausible events. In this case, properly comprehending or producing the sentence that describes the event requires syntactic knowledge of thematic role assignment. With active reversible sentences, processing is facilitated by the fact that word order is “canonical” — in subject-first languages, the “first noun bias” leads to assigning the role of agent to the first noun and that of theme to the second (hence, *The woman is hugging the man* if the woman is the doer) (Ferreira, 2003; Grodzinsky, 2000; Meyer et al., 2012). By contrast, the assignment of thematic roles in both comprehension and production alike is more demanding when a reversible sentence is in the passive voice, like in *The man is hugged by the woman*, as in this case word order is “non canonical”, due to a mismatch between superficial and deep sentence structure. In fact, in passives of subject-first languages, the first noun is the grammatical subject but takes the theme role, and the second noun is the agent complement but takes the agent role.

The results of research in persons with aphasia focusing on reversible sentence processing are fully consistent with the previous considerations. The comprehension of irreversible sentences was found to be substantially spared in these individuals, in the face of substantial problems with reversible stimuli (e.g., Berndt et al., 1996; Caplan & Futter, 1986; Caramazza & Zurif, 1976; Grodzinsky, 1986; Heilman & Scholes, 1976). Difficulties were usually more pronounced for passive than for active sentences (e.g., Brookshire & Nicholas, 1980; Meyer et al., 2012), even though thematic role reversals were observed also in response to actives (e.g., Berndt et al., 1996; Caramazza et al., 2005; Saffran et al., 1980). Difficulties with thematic analysis were observed irrespective of the canonical word order in the language examined. They were reported in SVO languages like English (Schwartz et al., 1980), Italian (e.g., Caramazza et al., 2005), German (e.g., Ditges et al., 2021), Dutch (e.g., Wassenaar & Hagoort, 2007), and Icelandic (e.g., Magnusdottir et al., 2013), but also in SOV languages like Japanese (Hagiwara & Caplan, 1990; Kinno et al., 2014), Korean (Kim et al., 2010), Persian (Shiani et al., 2019), and Malay (Aziz et al., 2020). In each case, sentences with non-canonical word order were affected more than sentences with canonical word order.

### Morphosyntactic Processes

In addition to thematic role assignment, the integrity of morphosyntactic processes is also a prerequisite for correct sentence elaboration. The complexity of morphological processes differs across languages. In English, they are critical for encoding active/passive sentence voice, subject/verb agreement, tense, pronouns, prepositions, etc. In morphologically richer languages, they can additionally indicate, for example, which noun is modified by an adjective, or specify the grammatical case*.*

Research on aphasia has repeatedly documented morphosyntactic difficulties. Agreement violations, morphologically incorrect nominal, adjectival and verbal forms, omissions and (less frequently) substitutions of auxiliary verbs, clitic pronouns, determiners, and prepositions have been attested countless times in the spontaneous speech of “agrammatic” speakers. Morphosyntactic errors in speech have been reported in a variety of languages, including English (Saffran et al., 1989), Italian (e.g., Miceli et al., 1989), German (e.g., Bates et al., 1988), French (e.g., Nespoulous et al., 1988), Turkish (e.g., Slobin, 1991), Dutch (e.g., Bastiaanse, 1995), and Persian (e.g., Nilipour & Raghibdoust, 2001). Various patterns of morphosyntactic impairment were also reported, in French (Tissot et al., 1973), English (Saffran et al., 1989), and Italian (Miceli et al., 1989). For extensive corpora of “agrammatic” speech errors in 26 languages, see Menn et al. (1990). Difficulties in comprehending morphosyntactic sentence features were reported less frequently (Dick et al., 2001; Fyndanis et al., 2013; Thompson & Mack, 2014).

Disorders of thematic and morphosyntactic processing typically co-occur in the same aphasic individual. In these cases, the nature of grammatical errors remains elusive. For example, when required to produce a passive reversible sentence like *The cook is pushed by the waiter*, a person with aphasia with severe morphosyntactic damage might make an error like *cook … push … waiter*, which may be interpreted as being thematically deviant, morphosyntactically deviant, or both. Even though thematic and morphosyntactic deficits are typically associated, dissociations have been described in single-case reports. Thematic role processing was selectively damaged both in production and in comprehension (Caramazza & Miceli, 1991; Miozzo et al., 2008; see also Maher et al., 1995), and morphosyntactic processes were selectively disrupted in speech (e.g., Miceli et al., 1983; Thompson et al., 2002; Tissot et al., 1973). These observations, albeit rare, suggest that the two sets of processes involve partly separable neural substrates, whose characterization has been at the forefront of research on language in the past several years.

### The Search for the Neural Correlates of Thematic Role Assignment and of Morphosyntactic Processing

Meta-analyses of neuroimaging studies in cognitively unimpaired participants show that the brain network subserving sentence processing extends from anterior to posterior fronto-temporoparietal regions of the left hemisphere (e.g., Demonet et al., 2005; Price, 2012; Walenski et al., 2019). The same neurofunctional correlates have been explored also in lesion studies with brain-damaged populations (e.g., Kristinsson et al., 2020; Magnusdottir et al., 2013; Pillay et al., 2017; Rogalsky et al., 2018; Thothathiri et al., 2012). In these investigations, deviant performance and lesion site are correlated both in individual cases and in large case series, in order to identify the brain structures critical for the cognitive process(es) under examination.

Lesion-symptom correlation studies on disorders of thematic role assignment in reversible sentences in persons with aphasia showed the involvement of posterior regions of the left hemisphere (Dronkers et al., 2004). The critical structures included the anterior and posterior portions of the superior and middle temporal gyri (Magnusdottir et al., 2013; Pillay et al., 2017; Rogalsky et al., 2018), the supramarginal and the angular gyrus (Meltzer et al., 2013; Thothathiri et al., 2012). These observations converge with findings of neuroimaging studies (Bornkessel et al., 2005; Mack et al., 2013; Meltzer-Asscher et al., 2013; Richardson et al., 2010) and transcranial magnetic stimulation investigations (Finocchiaro et al., 2015; Vercesi et al., 2020) in neurotypical subjects.

Strikingly, the same studies failed to reveal a similar association between reversible sentence processing and the left prefrontal regions. Traditionally, the posterior two-thirds of the inferior frontal gyrus (Brodmann Area 44/45) have been considered to be critical for language functions in general, and specifically for sentence processing (e.g., Caplan et al., 1996, 1998; Carreiras et al., 2012; Chang et al., 2018; Dapretto & Bookheimer, 1999; Friederici et al., 2003; Moro et al., 2001; Stromswold et al., 1996; Zurif et al., 1993). Due to the failure to correlate the left inferior frontal gyrus and sentence processing in lesion-symptom mapping studies, the functional role of this region has become a matter of debate. Different authors have claimed that the left inferior frontal gyrus is rather involved in extra-linguistic abilities (e.g., working memory, Baldo & Dronkers, 2006; Kaan & Swaab, 2002; Pettigrew & Hillis, 2014) or in domain-general processes (e.g., cognitive control, Novick et al., 2005; Rogalsky et al., 2018; decision making, Caplan et al., 2008).

Interestingly, however, the conclusions on the lack of correlation between the left inferior frontal gyrus and sentence comprehension were based on lesion-symptom mapping studies that focused almost exclusively on thematic role assignment and did not consider morphosyntactic processes, even though, as noted above, these are as critical as thematic analysis for sentence processing.

To help disentangle controversies regarding the neurofunctional correlates of sentence processing and more clearly delineate the functional role of the left prefrontal and temporoparietal regions, we systematically reviewed the literature on sentence processing difficulties in persons with aphasia. We considered lesion-symptom mapping studies that investigated impairments of thematic role assignment and/or of morphosyntactic processing. In our survey, we considered several dimensions: the neuropsychological tasks and measures used as correlation variables, the sentence processing component under scrutiny (i.e., thematic vs morphosyntactic), the response modality (i.e., sentence comprehension vs production), the etiologies underlying the aphasic disorders that resulted in poor sentence comprehension, and the languages examined. We also set out to complement the systematic literature review with a coordinate-based meta-analysis identifying the convergence of lesion-symptom correlations across studies. The pooled set of papers was sufficiently large to license the meta-analysis of the overall convergence effect. However, due to the scarcity of eligible studies, meta-analyses that contrasted the investigations focused specifically on thematic role assignment with those focused specifically on morphosyntactic processing were not supported by adequate statistical power (Eickhoff et al., 2016). They will be reported here, albeit for exploratory purposes only.

## Materials and Methods

### Systematic Literature Review: Study Selection

**Fig. 1:**
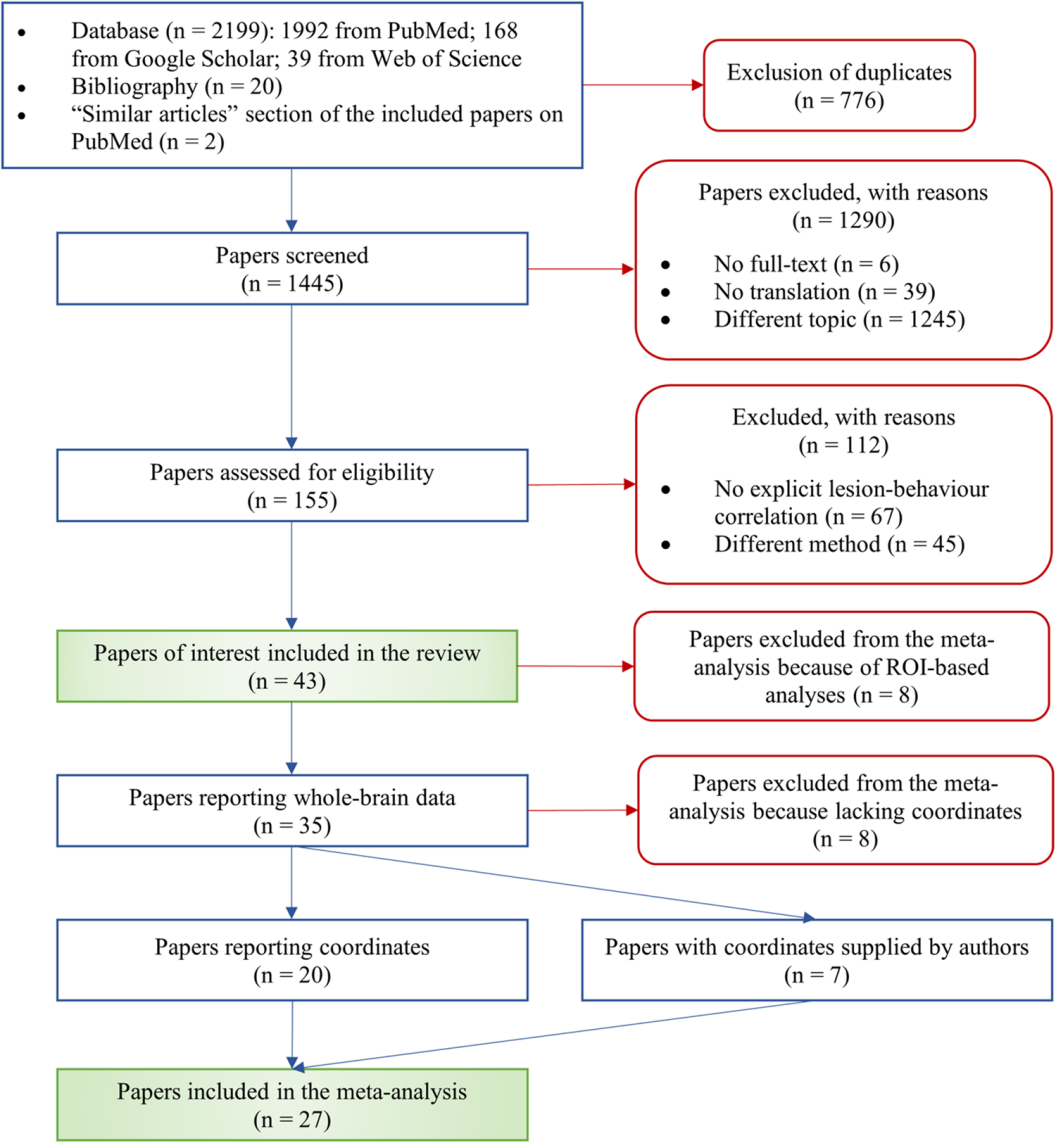
PRISMA flowchart for the literature search and selection process. Blue rectangles describe the selection steps, red frames specify the reasons for exclusion, while green shaded rectangles highlight the papers of interest for the systematic literature review and the coordinate-based meta-analysis, respectively

The literature search and selection of the papers were conducted according to the Preferred Reporting Items for Systematic Reviews and Meta-Analyses (PRISMA) guidelines (Page et al., 2021). All the papers published until September 2022 and available on PubMed, Google Scholar, and Web of Science were considered in the database search. Additional articles were identified through the references cited in the retrieved papers, through the “Similar articles” function in PubMed (Fig. 1).

Studies were identified based on the following criteria:

Inclusion criteria:

- Investigation of persons with aphasia with deficits in either thematic role assignment or morphosyntactic processing following stroke, primary progressive aphasia, glioma, or traumatic brain injury
- Collection of structural neuroimaging data through magnetic resonance imaging (MRI), computed tomography, computerized axial tomography, or positron emission tomography
- Correlation measures derived from cognitive tasks focusing on thematic role assignment and/or morphosyntactic processing
- Analysis of lesion-symptom correlations, using methods such as voxel-based lesion-symptom mapping (VLSM; also called voxel-based lesion-behavioral mapping-VLBM); support vector regression-lesion symptom mapping (SVR-LSM), or voxel-based morphometry (VBM)

Additional inclusion criteria for the meta-analysis:

- Report of standardized (either in Montreal Neurological Institute (MNI) or Talairach space) brain coordinates showing a correlation between brain lesions and language impairment;
- Whole-brain analyses, i.e., analyses that did not exclude any portions of the left-hemispheric cortical lesion volumes;

Exclusion criteria:

- No access to full text;
- Paper published in a language other than English, Italian, German, or French, and lacking translation;

Additional exclusion criteria for the meta-analysis:

- Regions of Interest (ROI)-based analyses

The following keyword combinations were entered in the literature search databases:

(aphas*) AND ((“brain damage”) OR (“brain injury”) OR (“brain lesion”) OR (“head damage”) OR (“head injury”) OR (stroke) OR (encephalitis) OR (lesion) OR (injury) OR (patient*) OR (“brain tumor*”) OR (glioma*) OR (“cerebral tumor*”) OR (“resective surgery”)) AND ((morphosynta*) OR (synta*) OR (morpholog*) OR (“inflectional morphology”) OR (inflection*) OR (agreement) OR (gramma*) OR (“syntactic disorder”) OR (“sentence structure”) OR (“phrase structure”) OR (“thematic roles”) OR (“thematic roles assignment”) OR (“verb argument structure”) OR (“reversible sentence*”) OR (reversibility) OR (“canonical sentence*”) OR (“non-canonical sentence*”) OR (passive*) OR (thema) OR (agent)) AND ((neuroimaging) OR (“magnetic resonance”) OR (neuroanatomic) OR (mri) OR (pet) OR (ct) OR (cat)) AND ((“voxel-based lesion symptom mapping”) OR (VLSM) OR (“lesion-symptom mapping”) OR (“lesion symptom mapping”) OR (LSM) OR (“lesion-behavior mapping”) OR (“lesion behavior mapping”) OR (LBM) OR (“voxel-wise lesion-symptom mapping”) OR (“voxel wise lesion symptom mapping”) OR (“voxel-based morphometry”) OR (VBM) OR (“support vector regression lesion-symptom mapping”) OR (“support vector regression lesion symptom mapping”) OR (SVR-LSM) OR (“multivariate lesion symptom mapping”) OR (“multivariate lesion-symptom mapping”) OR (MLSM) OR (“ROI-based lesion-symptom mapping”) OR (“ROI based lesion symptom mapping”) OR (RLSM) OR (“multivariate lesion-behavior mapping”) OR (“multivariate lesion behavior mapping”)).

Three authors (S.B., G.B., M.T.) contributed to the literature search and paper selection. All the authors double-checked data in case of discordance or uncertainty and resolved these cases through joint discussion.

A total of 2199 papers were initially retrieved through these keyword combinations. After the exclusion of duplicates (*n* = 776), there remained 1445 articles, which we screened based on inspection of their titles and abstracts. A total of 1290 papers was excluded at this step, because they either lacked the full text (*n* = 6); were written in a language other than English, Italian, German, or French and lacked translation (*n* = 39); and dealt with a different topic (*n* = 1245). The 155 remaining studies were further screened based on an inspection of their full texts. This led to exclude 112 papers that either lacked a lesion-symptom correlation (*n* = 67) or used a different method (*n* = 45). At the end of these selection steps, 43 eligible papers remained that focused on sentence processing, and more specifically on thematic role assignment or morphosyntactic processing considered in isolation, or on both thematic role assignment and morphosyntactic processing (criteria for classifying the studies are listed in Table 1). These 43 papers were included in our systematic literature review (Table 2). The behavioral tests and correlation variables, as well as the neuroimaging acquisition and analysis methods reported in the selected papers, are summarized in Tables 3 and 4.

**Table 1:** Definition of the criteria employed to classify studies based on the linguistic components they tested, with some relevant examples

Category	Description	Examples
TR	Task/measure specifically addressing TR, or involving some aspects of TR	TR reversal in a sentence-picture matching task including one TR reversal foil; TR reversal in a sentence-picture matching task involving two alternative foils (target picture, TR reversal foil, foil involving a different distractor (e.g., lexical))
MS	Task/measure specifically addressing MS, or involving some aspects of MS	Agrammatic errors (omission of function words and morphemes, simplification of sentence structure, wrong word forms, wrong word order) in a story retelling task; Mean length of utterances (morphemes) in a spontaneous speech production task
TR + MS	Task/measure addressing both TR and MS, or involving some aspects of both	Total score of TR and MS errors in a sentence-picture matching task with one alternative foil; Total score of TR and MS errors in a sentence-picture matching task involving two alternative foils or more

*TR* thematic role assignment, *MS* morphosyntactic processing

**Table 2:** Sample characteristics of the 43 studies included in our systematic literature review

Study	Lesion type	Sample size	Sex (Males)	Mean age (years (SD))	Mean education (years (SD))	Handedness (L)eft-handed/ (A)mbidextrous	Inclusion in meta-analysis
Amici et al., 2007	Atrophy	58	29	64.7 (8.8)	ns (ns)	0 L	No
Ash et al., 2013	Atrophy	30	ns	67.5 (9.4)	15.1 (2.9)	ns	Yes
Ash et al., 2015	Amyotrophic Lateral Sclerosis	10	8	60.4 (7.9)	13.9 (2.4)	ns	Yes
Baldo & Dronkers, 2007	Stroke (chronic, single, left hemisphere)	68	52	61.3 (ns)	15.2 (ns)	0 L	No
Borovsky et al., 2007	Stroke (chronic, single, left hemisphere)	50	ns	54 (12.0)	ns (ns)	5 L	Yes
Canu et al., 2019	Atrophy	27	11	69.3 (5.1)	11.4 (4.1)	0 L	Yes
Charles et al., 2014	Atrophy	46	ns	64.2 (8.6)	15.4 (3.3)	ns	Yes
DeLeon et al., 2012	Atrophy	43	23	66.8 (8.9)	15.8 (2.9)	3 L 2 A	Yes
den Ouden et al., 2019	Stroke (chronic, single *N* = 63/multiple *N* = 8, left-hemisphere, ischaemic)	62*	ns	59.6 (10.1)	ns (ns)	0 L	Yes
Dronkers et al., 2004	Stroke (chronic, single, left hemisphere (*n* = 64)/right hemisphere (*n* = 8))	72	56	62 (ns)	13.65 (ns)	0 L	No
Faroqi-Shah et al., 2014	Stroke (chronic, single, left hemisphere)	31	21	56.16 (ns)	ns (ns)	0 L	Yes
Grossman et al., 2013	Atrophy	12	ns	69.7 (11.7)	14.8 (2.6)	ns	Yes
Henry, 2009	Atrophy	11	5	72.1 (7.9)	15.9 (2.5)	1 L	Yes
Henseler et al., 2014	Stroke (single, left hemisphere, middle cerebral artery territory, ischaemic)	102	64	52.2 (10.5)	ns (ns)	ns	No
Kamminga et al., 2016	Atrophy (*n* = 42 (15 FTD-ALS, 27 PNFA)), Amyotrophic Lateral Sclerosis (*n* = 20)	62	37	62.8 (7.9)	12.8 (2.3)	ns	Yes
Kim et al., 2010	Stroke (acute, left hemisphere (*n* = 22)/right hemisphere (*n* = 13)/bilateral (*n* = 4), ischaemic)	39	26	61 (12.6)	10.5 (5.2)	0 L	Yes
Kinno et al., 2009	Glioma in the left frontal region: 11 with II grade tumors, 9 with III grade tumors, 1 with IV grade tumors	21*	12	35.4 (8.8)	ns (ns)	0 L	Yes
Kinno et al., 2014	Glioma (left premotor cortex (*n* = 7)/left opercular or triangular F3 (*n* = 7)/other left frontal regions (*n* = 7); II grade glioma (*n* = 10), III grade glioma (*n* = 11))	21*	12	34 (12.0)	ns (ns)	0 L	No
Kristinsson et al., 2020	Stroke (acute, single, left hemisphere)	104 (54 Icelandic; 50 USA)*	57 (29 Icelandic; 28 USA)	65.2 [67.1 Icelandic; 63.1 USA] (10 [11.1 Icelandic; 8.8 USA])	ns (ns)	ns	No
LaCroix et al., 2020	Stroke (chronic, left-hemisphere)	21	9	55 (13.8)	15.6 (2.8)	0 L	No
Lukic et al., 2017	Stroke (chronic, left-hemisphere)	40	26	59.4 (12.4)	16.1 (2.2)	1 L	Yes
Lukic et al., 2020	Stroke (chronic, single, left hemisphere, thrombo-embolic or hemorrhagic event)	76	50	58.6 (11.9)	15.9 (2.2)	10 L 2 A	Yes
Magnusdottir et al., 2013	Stroke (acute, single, left hemisphere, ischaemic)	50*	26	63.8 (11.5)	ns (ns)	ns	Yes
Matchin et al., 2020	Stroke (chronic, single, left-hemisphere, ischaemic)	61*	40	56.3 (12.9)	16 (2.6)	0 L	Yes
Matchin et al., 2021	Stroke (chronic, at least one, left hemisphere, ischaemic)	222 [Group 1: 130; Group 2: 92]*	143 [Group 1: 83; Group 2: 60]	59.4 (11) [Group 1: 60 (10.7) Group 2: 58.7 (11.3)]	15.4 (2.4) [Group 1: 15.4 (2.4) Group 2: 15.3 (2.4)]	ns	No
Matchin et al., 2022	Stroke (chronic, at least one, left hemisphere, ischaemic)	213 [Group 1: 121; Group 2: 92]*	139 [Group 1: 79; Group 2: 60]	59.2 (10.8) [Group 1: 59.6 (10.6) Group 2: 58.7 (11.1)	15.4 (2.3) [Group 1: 15.4 (2.3) Group 2: 15.3 (2.3)]	0 L	Yes
Meltzer et al., 2013	Stroke (chronic, single, left-hemisphere, ischaemic)	25	14	55.8 (10.9)	16.6 (2.6)	ns	Yes
Mesulam et al., 2021	Atrophy	62	33	65.1 (6.8)	ns (ns)	0 L	No
Mirman et al., 2019	Stroke (chronic, left hemisphere)	46*	27	ns (ns)	ns (ns)	0 L	Yes
Newhart et al., 2012	Stroke (acute, single, left hemisphere, ischaemic)	53	23	59.3 (15.2)	14 (3.4)	0 L	No
Peelle et al., 2008	Atrophy	29	ns	65.81 (12.2)	15.1 (2.17)	0 L	Yes
Pillay et al., 2017	Stroke (chronic, left hemisphere, focal encephalomalacia)	44	26	59.2 (12.6)	14.3 (3.0)	15 L 2 A	Yes
Riccardi et al., 2022	Stroke (chronic, left hemisphere)	60	42	57.9 (9.7)	15.1 (2.2)	7 L	No
Rogalski et al., 2011	Atrophy	31	11	63 (8.2)	16.2 (2.5)	0 L	No
Rogalsky et al., 2018	Stroke (*n* = 48, chronic, left hemisphere, focal lesion) Lobectomy (*n* = 18, left hemisphere, temporal)	66	39	54.4 (12.7)	ns (ns)	2 L 5 A	Yes
Sapolsky et al., 2010	Atrophy	23	9	64.3 (6.9)	15.5 (3.1)	ns	No
Sheppard et al., 2022	Stroke (left hemisphere, ischaemic) Each patient was tested both in the acute (within 6 days after stroke) and in the chronic (≥ 6 months post-stroke) stage	15	9	56 (15.9)	14.3 (2.8)	3 L	No
Thothathiri et al., 2012	Stroke (left hemisphere)	79*	ns	ns (ns)	ns (ns)	0 L	Yes
Tyler et al., 2011	Stroke (chronic, *n* = 13, left hemisphere) Post-surgical hematoma (*n* = 1, left hemisphere)	14	11	56 (ns)	ns (ns)	0 L	Yes
Wilson et al., 2010a	Atrophy	60	26	65.4 (7.6)	16.2 (2.7)	6 L	Yes
Wilson et al., 2011	Atrophy	27	15	66 (8.0)	ns (ns)	4 L	No
Wilson et al., 2016	Atrophy	51	27	65 (7.8)	16.7 (2.6)	9 L	Yes
Wu et al., 2007	Stroke (chronic, left hemisphere)	19	11	ns (ns)	ns (ns)	0 L	No

*ns* not specified

The following papers report biographical information referring to a larger sample than the one included in the analyses of interest for the present coordinate-based meta-analysis. We list here the number of participants in the total sample and that of participants included in the analysis of interest in the meta-analysis (as specified in the “Sample size” column):

Ash et al., 2013: total sample: 62 participants; analysis of interest conducted on: 30 participants

Ash et al., 2015: total sample: 26 participants; analysis of interest conducted on: 10 participants

Charles et al., 2014: total sample: 66 participants; analysis of interest conducted on: 46 participants

DeLeon et al., 2012: total sample: 46 participants; analysis of interest conducted on: 43 participants

den Ouden et al., 2019: total sample: 71 participants; analysis of interest conducted on: 62 participants

Grossman et al., 2013: total sample: 15 participants; analysis of interest conducted on: 12 participants

LaCroix et al., 2020: total sample: 25 participants; analysis of interest conducted on: 21 participants

Pillay et al., 2017: total sample: 51 participants; analysis of interest conducted on: 44 participants

An asterisk in the “Sample Size” column indicates a partially overlapping sample with that of other studies. More in details:

Kinno et al., 2014: partial sample overlap with Kinno et al., 2009 (*n* = 7)

Kristinsson et al., 2020: partial sample overlap with Magnusdottir et al., 2013 (*n* = 54)

Matchin et al., 2021: partial sample overlap with Den Ouden et al., 2019 (*n* = 82); Kristinsson et al., 2020 (*n* = 48); Matchin et al., 2020 (*n* = ns).

Matchin et al., 2022: partial sample overlap with Matchin et al., 2021 (*n* = ns)

Matchin et al., 2022 (Group 1; syntactic comprehension; *n* = 121): partial sample overlap with Den Ouden et al., 2019 (*n* = 47); Kristinsson et al., 2020 (*n* = 48); both Den Ouden et al., 2019 and Kristinsson et al., 2020 (*n* = 26)

Matchin et al., 2022 (Group 2; expressive agrammatism; *n* = 92): partial sample overlap with Den Ouden et al., 2019 (*n* = 39); Matchin et al., 2020 (*n* = 32); both Den Ouden et al., 2019 and Matchin et al., 2020 (*n* = 21)

Mirman et al., 2019: partial sample overlap with Thothathiri et al., 2012 (*n* = ns)

**Table 3:** Description of the tasks entering the different experiments

A				
Study	Task(s) for each statistical comparison considered in our literature review and meta-analysis Task name (bibliographical reference). *[Linguistic measure(s) of interest correlated with lesions.]*	Category	Modality	Language
Ash et al., 2013	Picture description Cookie theft picture (Goodglass & Kaplan, 1983). *[Composite grammaticality score: average of Z-scores for mean length of utterance in words, number of dependent clauses per utterance, and percent of well-formed sentences.]*	MS	P	English
Ash et al., 2015	Story telling "Frog, Where Are You?" picture book (Mercer Mayer, 1969). *[Percentage of utterances that were grammatically well-formed sentences.]*	MS	P	English
Borovsky et al., 2007	Spontaneous speech production Experimental task developed by Borovsky et al. (2007). *[Mean length of utterance in morphemes.]*	MS	P	English
Canu et al., 2019	Sentence anagram task Northwestern Anagram Test (Italian version, NAT-I; Canu et al., 2019). *[- Total NAT-I score computed from active and passive sentences, complex active sentences, object-extracted questions;* *- Non-canonical score computed from passive sentences, object-extracted questions.]*	TR+MS	P	Italian
Charles et al., 2014	Sentence-picture matching task Experimental task developed by Charles et al. (2014). *[Percent correct responses in sentence comprehension of:* *- Cleft sentences;* *- Center-embedded sentences.]*	TR	C	English
DeLeon et al., 2012	Sentence completion task Elicited production task (Goodglass et al., 1972). *[Accuracy on targeted structures: imperative, declarative with 3*^*rd*^* person present agreement, yes or no interrogative in past tense, wh-interrogative declarative in past tense, future, declarative with embedded small clause, passive in past tense, and comparative sentences]*	MS	P	English
den Ouden et al., 2019	Picture description Cookie theft picture (Kertesz, 2007). *[Categorical variable indicating patients with an “agrammatic pattern of morphosyntactic reduction”: omission and substitution of grammatical morphemes (verb inflection: tense and agreement errors; plural markers), articles, prepositions.]*	MS	P	English
	Primed sentence production task Sentence Production Priming Test (SPPT; Cho-Reyes & Thompson, 2012). Sentence-picture matching task Sentence Comprehension Test (SCT; Cho-Reyes & Thompson, 2012). Verb argument structure production in sentence context Argument Structure Production Test (ASPT; Cho-Reyes & Thompson, 2012). *[Total score computed from active and passive sentences, subject- and object-extracted wh-questions, subject and object relative clauses, and verb forms that included one- two- three-argument verbs.]*	TR+MS	C/P	
Faroqi-Shah et al., 2014	Picture description Picture Description (PAL, subtest 14; Caplan, unpublished). *[Accuracy on target syntactic structure conveying thematic roles and attribution of modification computed from active and passive sentences, dative active and passive, subject and object relative sentences (covariates: nonword repetition task performance and picture naming task performance).]*	TR+MS	P	English
Grossman et al., 2013	Picture description Cookie theft picture (Goodglass & Kaplan, 1983). *[Percentage of grammatically well-formed sentences.]*	MS	P	English
Henry, 2009	Primed sentence production task Sentence Production Priming Test (SPPT; Cho-Reyes & Thompson, 2012). Sentence-picture matching task Sentence Comprehension Test (SCT; Cho-Reyes & Thompson, 2012). Verb production task Verb Naming Test (VNT; Cho-Reyes & Thompson, 2012). Verb-picture matching task Verb Comprehension Test (VCT; Cho-Reyes & Thompson, 2012). Sentence production with arguments Argument Structure Production Test (ASPT; Cho-Reyes & Thompson, 2012). *[Syntactic composite score: average of correct percent in active and passive sentences, subject- and object-extracted wh-questions, subject and object relative clauses. Verb forms included one- two- three-argument verbs.]*	TR+MS	C/P	English
Kamminga et al., 2016	Sentence–picture matching task Abbreviated-TROG (selection of 2 out of 4 sentences for each block; Bishop, 1989). *[Abbreviated-TROG total score computed from different types of sentences with single nouns, verbs, and adjectives, combined in various ways.]*	TR+MS	C	English
Kim et al., 2010	Question comprehension task Korean Syntactic Comprehension Test (KSCT; Kim et al., 2010). *[KSCT total score computed from different syntactic morphemes from those in the original sentence and the constant or altered word order was used in the question.]*	TR	C	Korean
Kinno et al., 2009	Sentence-picture plausibility task Experimental task developed by Kinno et al. (2009). *[- Error rates on passive (non-canonical and subject-initial passives) sentences (covariate: active (canonical and subject-initial actives) sentence score);* *- Error rates on scrambled (non-canonical and object-initial scrambled) sentences (covariate: active sentence score).]*	TR	C	Japanese
Lukic et al., 2017	Sentence-picture matching task Sentence Comprehension Test (SCT; Cho-Reyes & Thompson, 2012). *[SCT score computed from active and passive sentences, subject- and object-extracted wh-questions, subject and object relative clauses.]*	TR	C	English
Lukic et al., 2020	Sentence-picture matching task Sentence Comprehension Test (SCT; Cho-Reyes & Thompson, 2012). *[- Total SCT score computed from canonical sentences (active, subject-extracted wh-questions, subject relative clauses) and non-canonical sentences (passive, object-extracted wh-questions, object relative clauses) (covariate: verb comprehension score);* *- Total SCT score (covariate: sentence production score);* *- Non-canonical sentence score (covariate: canonical sentence score).]*	TR	C	English
	Primed sentence production task Sentence Production Priming Test (SPPT; Cho-Reyes & Thompson, 2012). *[- Total SPPT score computed from canonical sentences (active, subject-extracted wh-questions, subject relative clauses) and non-canonical sentences (passive, object-extracted wh-questions, object relative clauses) (covariate: verb production score);* *- Total SPPT score (covariate: sentence comprehension score);* *- Non-canonical sentence score (covariate: canonical sentence score).]*	TR+MS	P	
Magnusdottir et al., 2013	Sentence-picture matching task Experimental task developed by Magnusdottir (2005). *[- Non-canonical sentence score computed from passive sentences, cleft sentences with object gap, referential wh-question with object gap and main verb;* *- Non-canonical sentence score (covariate: canonical (active declaratives, cleft sentences with subject gap, referential wh-question with subject gap and main verb) sentence score).]*	TR	C	Icelandic
Matchin et al., 2020	Story telling "Cinderella" story picture book (Grimes, 2005). *[- Agrammatism score: errors on functional word, morpheme omission, reduced sentence complexity (covariate: words per minute score);* *- Paragrammatism score: grammatical errors or sporadic omissions with a general presence of functional elements (covariate: words per minute score).]*	MS	P	English
Matchin et al., 2022	Picture description Cookie theft picture (Kertesz, 2007). Story telling "Cinderella" story picture book (Grimes, 2005). *[Expressive agrammatism score: errors on functional word, morpheme omission, reduced sentence complexity.]*	MS	P	English
	Sentence-picture matching task Experimental task developed by Magnusdottir (2005). Sentence Comprehension Test (SCT; Cho-Reyes & Thompson, 2012). *[Syntactic comprehension measure: the average performance on object-extracted clefts, object-extracted relative clauses, and object-extracted Wh-questions (covariate: performance on simple, semantically reversible active sentences).]*	TR	C	
Meltzer et al., 2013	Sentence-picture matching task Experimental task developed by Meltzer & Braun (2011). *[Syntactic comprehension score computed from reversible non-complex sentences.]*	TR	C	English
Mirman et al., 2019	Story retelling "Cinderella" story or familiar fairy tale *[Proportion of words per sentence.]*	MS	P	English
Peelle et al., 2008	Question comprehension task Experimental task developed by Grossman et al. (1996). *[- Sentence comprehension score computed from subject–verb–object sentences, subject and object-relative embedded clauses;* *- Sentence comprehension score (covariate: working memory task performance).]*	TR	C	English
Pillay et al., 2017	Sentence-video plausibility task Auditory Sentence Comprehension (ASC; Westbury, 2015). *[ASC accuracy score (covariate: picture naming task performance).]*	TR	C	English
Rogalsky et al., 2018	Sentence–picture matching task Subject-relative, Object-relative, Active, and Passive (SOAP; Love & Oster, 2002). Sentence plausibility judgement task Plausibility judgment task (Rogalsky, 2018). *[- Total SOAP score computed from canonical (actives, subject-relative) and non-canonical sentences (passives, object relatives);* *- Non-canonical sentence SOAP score (covariate: canonical sentence score);* *- Total plausibility judgements score computed from passive, active, subject-relative, and object-relative;* *- Non-canonical sentence plausibility judgements score (covariate: canonical sentence score).]*	TR	C	English
Thothathiri et al., 2012	Sentence-picture matching task Sentence-picture matching task from the Philadelphia Comprehension Battery (PCB; Saffran et al., 1988). *[- Total score computed from canonical sentence (actives, subject relative clauses) and non-canonical sentence (passives, object relative clauses);* *- Total score (covariate: nonword repetition task performance);* *- Total score (covariate: rhyme probe spans task performance);* *- Non-canonical sentence score.]*	TR	C	English
Tyler et al., 2011	Sentence-picture matching task Experimental task developed by Ostrin & Tyler (1995). *[Role reversal errors computed from active and passive sentences.]*	TR	C	English
	Sentence plausibility judgement task Experimental task developed by Tyler et al. (2011). *[Unacceptable judgements computed from ambiguous sentences (agreement violation) (covariate: unambiguous sentence score).]*	TR+MS	C	
Wilson et al., 2010a	Picture description Picnic picture (Kertesz, 1982). *[Syntax principal component: proportion of words in sentences, ungrammatical sentence, e.g., missing determiners and inflections.]*	MS	P	English
Wilson et al., 2016	Sentence-picture matching task Experimental task developed by Wilson et al. (2010b). *[Accuracy score computed from short active and passive sentences, long easy, long, medium and long hard sentences.]*	TR	C	English
B				
Study	Task(s) for each statistical comparison considered in our literature review Task name (bibliographical reference). *[Linguistic measure(s) of interest correlated with lesions.]*	**Category**	**Modality**	**Language**
Amici et al., 2007	Sentence-picture matching task Curtiss-Yamada Comprehensive Language Evaluation – Receptive (CYCLE-R)11 subtests(Curtiss & Yamada, Unpublished Test, UCLA﻿). *[- Total CYCLE-R score computed from simple declaratives, possession, active voice word order, double embedding, agentless passive, agentive passive, subject relative clauses, object clefting, object (o-s) relative clauses, negative passive, and object (o-o) relative clauses;* *- Multiclausal relative sentence;* *- Common effect of multiclausal relative sentence comprehension;* *- Multiclausal relative sentence comprehension (covariate: verbal working memory performance).]*	TR	C	English
Baldo & Dronkers, 2007	Sentence-picture matching task Curtiss-Yamada Comprehensive Language Evaluation (CYCLE) subtests 4.2 and 5.6 (Curtiss & Yamada, Unpublished Test, UCLA. *[Total CYCLE subtests score computed from active/passive sentences.]*	TR	C	English
Dronkers et al., 2004	Sentence-picture matching task (Curtiss-Yamada Comprehensive Language Evaluation – Receptive (CYCLE-R)11 subtests (Curtiss & Yamada, Unpublished Test, UCLA﻿). *[Total CYCLE-R score computed from simple declaratives, possession, active voice word order, double embedding, agentless passive, agentive passive, subject relative clauses, object clefting, object (o-s) relative clauses, negative passive, and object (o-o) relative clauses.]*	TR	C	English
Henseler et al., 2014	Semi-standardized interview Aachen Aphasia Test (AAT; Huber et al., 1984). *[Correctness of grammar and syntactic complexity, including scoring of morphosyntactic errors.]*	MS	P	German
Kinno et al., 2014	Sentence-picture plausibility task Experimental task developed by Kinno et al., (2009). *[- Scrambled (non-canonical and object-initial scrambled) sentence score (covariates: active (canonical and subject-initial active score) and passive (non-canonical and subject-initial passives) sentence scores);* *- Passive and scrambled sentence scores (covariate: active sentence score).]*	TR	C	Japanese
Kristinsson et al., 2020	Sentence-picture matching task Experimental task developed by Magnusdottir (2005). *[- Canonical sentence score computed from active declaratives, cleft sentences with subject gap, referential wh-question with subject gap and main verb);* *- Non-canonical sentence score computed from passive sentences, cleft sentences with object gap, referential wh-question with object gap and main verb;* *- Non-canonical sentence score (covariate: canonical sentence score).]*	TR	C	Icelandic, English
LaCroix et al., 2020	Sentence-picture matching task Experimental task developed by Wilson et al. (2010b). *[Accuracy score computed from non-canonical sentences with prosody manipulations.]*	TR	C	English
Matchin et al., 2021	Picture description Cookie theft picture (Kertesz, 2007). Story telling "Cinderella" story picture book (Grimes, 2005). *[Agrammatism computed as the systematic simplification of sentence structure and omission of function words and morphemes.]*	MS	P	English
	Sentence-picture matching task Sentence Comprehension Test (SCT; Cho-Reyes & Thompson, 2012) Sentence-picture matching task Experimental task developed by Magnusdottir, (2005). *[- Non-canonical sentence comprehension score computed from the SCT (passives with a by-phrase, object-extracted Wh-questions, object-relatives);* *- Non-canonical sentence comprehension score computed from Magnusdottir’s task (2005; passives with a by-phrase, object-extracted Wh-questions, object clefts).]*	TR	C	
Mesulam et al., 2021	Story telling "Cinderella" story picture book (Grimes, 2005). *[Grammatical accuracy computed from noun morphology, verb morphology, argument structure, word order.]*	MS	P	English
	Primed sentence production and sentence anagram task Sentence Production Priming Test (SPPT; Cho-Reyes & Thompson, 2012). Northwestern Anagram Test (NAT; Weintraub et al., 2010). *[NAT-NAVS composite score: average of performance on SPPT and NAT tests.]*	TR+MS	P	
Newhart et al., 2012	Sentence-picture matching task Sentence Picture Matching (SPM; ns). Sentence-action matching Enactment (ns). *[Asyntactic comprehension score computed as performance not significantly above chance level of accuracy on passive reversible sentences on at least one test (SPM or enactment), and ≥ 10 percentage points lower accuracy on passive compared to active sentences and object-cleft compared to subject-cleft sentences, and ≥ 10 percentage points lower accuracy on reversible compared to irreversible sentences.]*	TR	C	English
Riccardi et al., 2022	Sentence plausibility judgement task Auditory Sentence Sensibility test (Fernandino et al., 2013). *[Auditory sentence sensibility score (covariate: forward digit span test performance).]*	TR	C	English
Rogalski et al., 2011	Story telling "Cinderella" story picture book (Grimes, 2005). *[Mean length of utterance in words.]*	MS	P	English
	Sentence anagram task Northwestern Anagram Test (NAT; subtest of 10 item from) (Weintraub et al., 2010). *[Subtest-NAT score computed from subject- and object-extracted who-question production.]*	TR+MS	P	
Sapolsky et al., 2010	Semi-structured interview Progressive Aphasia Severity Scale (PASS) (Sapolsky et al., 2010). *[Syntax and grammar accuracy score computed on word forms, functor words, word order.]*	MS	P	English
Sheppard et al., 2022	Sentence-picture matching task Subject-relative, Object-relative, Active, and Passive (SOAP; Love & Oster, 2002). *[- Accuracy score on non-canonical sentences (passives, object-relative);* *- Asyntactic comprehension pattern score: mean accuracy of noncanonical minus mean accuracy of canonical sentences (actives, subject-relative).]*	TR	C	English
Wilson et al., 2011	Sentence-picture matching task Experimental task developed by Wilson et al. (2010b). Sentence-picture matching task Curtiss-Yamada Comprehensive Language Evaluation (CYCLE) (Curtiss & Yamada, Unpublished Test, UCLA﻿). Sentence completion task Experimental task developed by Goodglass et al. (1972). Spontaneous speech and picture description ns (ns). *[Total score on comprehension and production tasks.]*	TR+MS	C/P	English
Wu et al., 2007	Sentence-picture matching task Experimental task adapted from Saffran et al. (1980) and from Schwartz et al. (1980) *[Thematic role errors on active sentences.]* Sentence-picture matching task Experimental task developed by Wu et al. (2007). *[Ability to select the correct pictorial representation of the consequence of spoken active sentences, with some alternatives targeting thematic roles comprehension.]*	TR	C	English

A, Studies included in both the coordinate-based meta-analysis and the systematic literature review; B, studies included only in the systematic literature review

*TR* thematic role assignment, *MS* morphosyntactic processing, *C* comprehension, *P* production

**Table 4:** Methods of acquisition and type of lesion-symptom analysis of the linguistic processes of interest

Study	Acquisition method	Analysis method	Anatomical space	Whole brain or ROI analysis	Lesion volume covariate
A					
Ash et al., 2013	MRI	VBM	MNI	Whole brain (all areas of gray matter disease)	No
Ash et al., 2015	MRI	VBM	MNI	Whole brain (all areas of gray matter disease)	No
Borovsky et al., 2007	MRI/CT	VLSM	Talairach	Whole brain	No
Canu et al., 2019	MRI	VBM	MNI	Whole brain	No
Charles et al., 2014	MRI	VBM	MNI	Whole brain	No
DeLeon et al., 2012	MRI	VBM	MNI	Whole brain analysis first assessed that no regions outside the mask were significantly associated with the linguistic measure in a non-permuted analysis. A subsequent analysis then applied a mask including left hemisphere perisylvian language areas, based on the Tzourio-Mazoyer et al. (2002) atlas (left inferior frontal gyrus (pars opercularis and triangularis), rolandic operculum, superior temporal gyrus, supramarginal gyrus)).	No
den Ouden et al., 2019	MRI	VLSM	MNI	Whole brain	Yes
Faroqi-Shah et al., 2014	MRI	VLSM	MNI	Whole brain	No
Grossman et al., 2013	MRI	VBM	Talairach	Whole brain (all areas of gray matter disease)	No
Henry, 2009	MRI	VBM	MNI	Whole brain	No
Kamminga et al., 2016	MRI	VBM	MNI	Whole brain	No
Kim et al., 2010	MRI	VLSM	MNI	Whole brain	No
Kinno et al., 2009	MRI	VLSM	MNI	Whole brain	No
Lukic et al., 2017	MRI	VLSM	MNI	Left hemisphere	Yes
Lukic et al., 2020	MRI	VLSM	MNI	Left hemisphere	Yes
Magnusdottir et al., 2013	MRI	VLSM	MNI	Whole brain	Yes
Matchin et al., 2020	MRI	VLSM	MNI	Whole brain	Yes
Matchin et al., 2022	MRI	VLSM	MNI	Whole brain	Yes
Meltzer et al., 2013	MRI	VLSM	MNI	Whole brain	Controlled by separate correlation analyses
Mirman et al., 2019	MRI (*n* = 30) / CT (*n* = 16)	SVR-LSM	MNI	Whole brain	Yes
Peelle et al., 2008	MRI	VBM	Talairach	Whole brain (all areas of gray matter disease)	No
Pillay et al., 2017	MRI	VLSM	MNI	Whole brain	Yes
Rogalsky et al., 2018	MRI (*n* = 62) / CT (*n* = 4)	VLSM	Talairach	Whole brain	Yes
Thothathiri et al., 2012	MRI (*n* = 43) / CT (*n* = 36)	VLSM	MNI	Whole brain	No
Tyler et al., 2011	MRI	VLSM	MNI	Whole brain	No
Wilson et al., 2010a	MRI	VBM	MNI	Whole brain (all areas of gray matter disease)	No
Wilson et al., 2016	MRI	VBM	MNI	Whole brain	No
B					
Amici et al., 2007	MRI	VBM	MNI	ROIs: left inferior and middle frontal gyri, left superior and middle temporal gyri, left inferior parietal lobule	No
Baldo & Dronkers, 2007	MRI/CT	VLSM	ns	Left hemisphere	No
Dronkers et al., 2004	MRI/CT	VLSM	Talairach	Whole brain	No
Henseler et al., 2014	MRI	VLSM	MNI	Whole brain	No
Kinno et al., 2014	MRI	VLSM	MNI	Whole brain	No
Kristinsson et al., 2020	MRI/CT	RLSM	MNI	ROIs: inferior frontal gyrus (pars opercularis and pars triangularis), supramarginal gyrus, angular gyrus, superior temporal gyrus, pole of superior temporal gyrus, middle temporal gyrus, pole of middle temporal gyrus, posterior superior temporal gyrus, posterior middle temporal gyrus	Yes
LaCroix et al., 2020	MRI	RLSM	MNI	ROIs: posterior half of the left middle frontal gyrus, left inferior frontal gyrus (pars opercularis and pars triangularis), left posterior superior temporal gyrus, left supramarginal gyrus, left angular gyrus	Yes
Matchin et al., 2021	MRI	VLSM	MNI	Whole brain	Yes
Mesulam et al., 2021	MRI	General linear model	MNI	ROIs: posterior part of the middle frontal gyrus, premotor cortex posterior to the inferior frontal gyrus), superior frontal gyrus, inferior frontal gyrus (pars opercularis), inferior frontal gyrus (junction of the pars triangularis with the pars orbitalis)	No
Newhart et al., 2012	MRI	RLSM	MNI	ROIs: Brodmann Areas 6, 10, 11, 18, 19, 20, 21, 22, 37, 38, 39, 40, 44, 45	No
Riccardi et al., 2022	MRI	RLSM	Talairach	ROIs: left inferior frontal gyrus (pars opercularis), left inferior frontal gyrus (pars triangularis), middle temporal gyrus pole, superior temporal gyrus pole, anterior portion of the inferior temporal gyrus, posterior middle temporal gyrus, superior temporal gyrus, supramarginal gyrus, angular gyrus	No
Rogalski et al., 2011	MRI	General linear model	ns	Whole brain	No
Sapolsky et al., 2010	MRI	VBM	ns	Whole brain	No
Sheppard et al., 2022	MRI	RLSM (LASSO regression)	ns	ROIs: inferior frontal gyrus (pars triangularis and pars opercularis, superior temporal gyrus, middle temporal gyrus, posterior superior temporal gyrus, temporal pole, angular gyrus, supramarginal gyrus, superior longitudinal fasciculus, inferior fronto-occipital fasciculus)	Yes
Wilson et al., 2011	MRI	VBM	MNI	ROIs: left inferior frontal gyrus, left inferior frontal cortex	No
Wu et al., 2007	MRI/CT	VLSM	ns	Whole brain	No

A, Studies included in both the coordinate-based meta-analysis and the systematic literature review; B, studies included only in the systematic literature review All the papers used whole brain coverage for the neuroimaging acquisition

*MRI* magnetic resonance imaging, *CT* computed tomography, *VBM* voxel-based morphometry; *VLSM* voxel-based lesion-symptom mapping, *RLSM* region-based lesion-symptom mapping, *SVR-LSM* support vector regression-lesion symptom mapping, *MNI* Montreal Neurological Institute, *ns* not specified

The 43 papers retained for review were further screened for eligibility for our coordinate-based meta-analysis. This led to excluding 8 studies that employed ROI-based rather than whole-brain analyses. Of the remaining 35 papers, 15 did not report standardized brain coordinates, but for 7 of these, the coordinates were obtained via direct request to the authors. The remaining 8 papers, for which standardized brain coordinates could not be obtained, were excluded. As a result of this selection procedure, we were left with 27 eligible papers for our meta-analysis (Table 2). Our selection of studies is subject to several risks of bias, including the following: mean age and mean education level of the study sample, which are not always reported (Table 2); handedness characteristics, which are heterogeneous (Table 2); etiology of the lesions, which differs within and between studies (Table 2); the inclusion of partially overlapping samples in some of the studies (Table 2); the heterogeneity of stimuli and tasks used to assess language abilities (Table 3); the combination of different neuroimaging techniques, namely either magnetic resonance imaging or computed tomography (Table 4); the variability of lesion-symptom correlation analysis methods across studies (Table 4); the bias inherent in ROI analyses, that implicitly leaves out brain regions (Table 4; note that studies resorting to ROI analysis were not included in our meta-analyses); the inconsistent consideration of the influence of lesion volume on lesion-symptom correlations (Table 4). We discuss these risks of bias more extensively in the “Discussion” and the “Limitations” sections.

### Meta-analysis

The coordinate-based meta-analysis relied on the revised activation likelihood estimation (ALE) algorithm implemented into the GingerALE software (version 3.0.2; https://brainmap.org/ale/). The ALE algorithm allows one to estimate the anatomical convergence of the standardized brain coordinates reported across a set of experiments.

Of the 27 selected papers, 23 reported standardized coordinates in the MNI space, and 4 in the Talairach space (Table 4). Talairach coordinates were transformed (Muller et al., 2018) into the MNI space using the icbm2tal algorithm implemented in the GingerALE toolbox.

The 27 papers included a total of 31 different experiments (i.e., statistical comparisons, Table 3). They were grouped in three sets, based on their linguistic focus (Tables 1, and 5). Set 1 included experiments focusing on thematic role assignment (“thematic role set”). Set 2 included experiments testing morphosyntactic processing abilities (“morphosyntactic set”). Set 3 included experiments using tasks that addressed both thematic role and morphosyntactic processing (“thematic role + morphosyntactic set”).

**Table 5:** Inclusion (indicated by “x”) of experiments in the reported meta-analyses (m.a., as numbered in the “Meta-analysis” section of the manuscript and in Table 6), and their categories and modalities based on the linguistic component they tested

Study	Category	Modality	m.a.1	m.a.2	m.a.3	m.a.4	m.a.5	m.a.6
Ash et al., 2013	MS	P	x	x		x	x	
Ash et al., 2015	MS	P	x	x		x	x	
Borovsky et al., 2007	MS	P	x	x		x	x	
Canu et al., 2019	TR + MS	P	x	x	x			
Charles et al., 2014	TR	C	x		x	x		x
DeLeon et al., 2012	MS	P	x	x		x	x	
den Ouden et al., 2019	MS	P	x*	x*		x	x	
	TR + MS	C/P			x			
Faroqi-Shah et al., 2014	TR + MS	P	x	x	x			
Grossman et al., 2013	MS	P	x	x		x	x	
Henry, 2009	TR + MS	C/P	x	x	x			
Kamminga et al., 2016	TR + MS	C	x	x	x			
Kim et al., 2010	TR	C	x		x	x		x
Kinno et al., 2009	TR	C	x		x	x		x
Lukic et al., 2017	TR	C	x		x	x		x
Lukic et al., 2020	TR	C	x*		x*	x		x
	TR + MS	P		x				
Magnusdottir et al., 2013	TR	C	x		x	x		x
Matchin et al., 2020	MS	P	x	x		x	x	
Matchin et al., 2022	MS	P	x	x		x	x	
	TR	C	x		x	x		x
Meltzer et al., 2013	TR	C	x		x	x		x
Mirman et al., 2019	MS	P	x	x		x	x	
Peelle et al., 2008	TR	C	x		x	x		x
Pillay et al., 2017	TR	C	x		x	x		x
Rogalsky et al., 2018	TR	C	x		x	x		x
Thothathiri et al., 2012	TR	C	x		x	x		x
Tyler et al., 2011	TR	C	x*		x*	x		x
	TR + MS	C		x				
Wilson et al., 2010a	MS	P	x	x		x	x	
Wilson et al., 2016	TR	C	x		x	x		x

Note that the study by Matchin et al. (2022) reported two independent experiments in separate patient cohorts (see Table 1). Two non-independent experiments were included in each study by den Ouden et al., (2019, Lukic et al. (2020), and Tyler et al. (2011). When both experiments were included in a meta-analysis, they were pooled as a single experiment (as indicated by an asterisk)

*TR* thematic role assignment, *MS* morphosyntactic processing, *C* comprehension task, *P* production task, *m.a.* meta-analysis

Distinct analyses were carried out to investigate the neurofunctional correlates of sentence processing components (Table 5):

- Meta-analysis (1) thematic role assignment or morphosyntactic processing (all the papers retained for analysis)
- Meta-analysis 2) thematic role assignment (“thematic role set” and “thematic role + morphosyntactic set”)
- Meta-analysis 3) morphosyntactic processing (“morphosyntactic set” and “thematic role + morphosyntactic set”)
- Meta-analysis 4) thematic role assignment and morphosyntactic processing (conjunction of “thematic role set” and “morphosyntactic set”)
- Meta-analysis 5) thematic role assignment — morphosyntactic processing (“thematic role set” minus “morphosyntactic set”)
- Meta-analysis 6) morphosyntactic processing — thematic role assignment (“morphosyntactic set” minus “thematic role set”)

Analysis 1 encompassed all 31 experiments (which narrowed to 28 when non-independent studies were pooled; see Table 5 and Muller et al., 2018), thus meeting the minimum sample size (*n* = 17–20) recommended in order to reach adequate statistical power in coordinate-based meta-analyses (Eickhoff et al., 2016). Sample sizes for analyses 2–6 were at or below the recommended minimum. The results of these analyses will be reported for merely exploratory purposes.

The results of analyses 1–4 were considered significant by declaring a cluster-level *p* < 0.05 threshold with family-wise error type correction for multiple comparisons, with an uncorrected *p* < 0.001 cluster-forming threshold and 1000 permutations. For analyses 5–6, given the unavailability of family-wise error rate (FWE) correction for direct comparisons between sets in GingerALE software, the false discovery rate (FDR) correction was adopted, declaring a *p* < 0.05 threshold based on 1000 permutations (for exploratory purposes, the threshold was also lowered to *p* < 0.05 uncorrected). The anatomical localization of the significant clusters was mapped via the AAL Toolbox (Tzourio-Mazoyer et al., 2002).

## Systematic Literature Review: Results

The systematic review of the published literature relies on 43 papers of interest (Tables 2, 3, and 4).

### Morphosyntactic Processing and Thematic Role Assignment Deficits in Persons with Aphasia: Main Features of the Papers Retained for Analysis

The 43 papers reported a total of 50 experiments investigating either thematic role assignment or morphosyntactic processing considered in isolation, or both (Table 3). More in detail, 25 experiments (50%) were on thematic role assignment and 15 (30%) on morphosyntactic processing. The remaining 10 experiments (20%) considered both thematic role assignment and morphosyntactic processing. None of the experiments contrasted the two processes directly.

#### Processing Modality

Most experiments (Table 3) assessed sentence comprehension (27/50, or 54%). Other experiments focused on production (20/50, or 40%) or on both modalities (3/50, or 6%).

#### Targeted Processing Modality and Ability

A more substantial imbalance emerges when not only modality but also the process targeted in the various experiments is considered. All the experiments that selectively addressed thematic role assignment impairment (*n* = 25) focused on comprehension, and all those only concerned with morphosyntactic deficits focused on production (*n* = 15). The remaining 10 experiments considered both thematic and morphosyntactic processes. They investigated production in 5 cases, comprehension in 2, and both modalities in 3 (Table 3).

#### Stimuli and Tasks Used to Assess the Targeted Ability/Abilities(Table 3)

Except for two experiments by Kinno et al., (2009, 2014) that used written stimuli, thematic role comprehension was studied by means of auditory stimuli. Most experiments on thematic role comprehension (19/25, 76%) included sentence-picture matching tasks (i.e., the participant hears a sentence and must choose the corresponding picture from an array of 2–4) or sentence-picture verification tasks (i.e., the participant is presented with a sentence and a picture/movie and must decide if they match). The remaining experiments on thematic role comprehension (6/25, 24%) included varied paradigms, e.g., sentence acceptability tasks, in which participants must decide if a whole sentence (Riccardi et al., 2022) or a constituent that completes a sentence (Tyler et al., 2011) makes common sense. Stimulus materials also changed across tasks and experiments. Both canonical and non-canonical sentences were used in most cases. The canonical structures employed more frequently were actives, subject-relatives, and subject-extracted Wh-questions. The non-canonical structures used most frequently were passives, object-relatives, object-extracted Wh-questions, and cleft sentences with object gaps. Only declarative sentences with canonical word order were employed by Wu et al. (2007).

All the experiments that selectively tackled morphosyntactic processing investigated spoken production, except Sapolsky et al. (2010), who focused on written output. Most experiments aimed at collecting samples of narrative speech in order to measure difficulties on a variety of linguistic dimensions, such as errors in free-standing and bound grammatical morphemes, reduced syntactic complexity, reduced mean length of utterance in morphemes, and incorrect word order. Picture description was used most frequently (in 10/15 experiments that considered only morphosyntactic processing, 66.7%). In some experiments, participants were asked to narrate a fairy tale. In three studies (Borovsky et al., 2007; Henseler et al., 2014; Sapolsky et al., 2010), speech corpora were collected during interviews. DeLeon et al. (2012) administered sentence completion tasks.

The remaining 10 experiments evaluating both thematic role assignment and morphosyntactic processes included a variety of tasks. In production, for example, 4 experiments included the Sentence Production Priming Test of the Northwestern Assessment of Verbs and Sentences (NAVS, Cho-Reyes & Thompson, 2012), in which a syntactic prime is presented as a template and the participant is asked to produce a sentence with the same structure. Agrammatic production was investigated also by a sentence anagram task (Northwestern Anagram Test; Thompson et al., 2012) or by asking participants to produce sentences containing the inflected forms of words whose citation form was presented in writing (Northwestern Anagram Test-Italian; Canu et al., 2019).

#### Languages

Regardless of whether focused on thematic role assignment or morphosyntactic processing, most experiments (43/50, or 86%) were conducted in English. Of the remaining experiments, two each were conducted in Japanese and Icelandic, and one each in Italian, German, and Korean (Table 3).

#### Etiology

Overall (Table 2), the 43 selected papers analyzed sentence processing deficits in 2256 participants, most of whom (1614, 71.5%) were stroke survivors in the chronic phase (1353/1614, 83.8%) or, much less frequently, in the acute phase of the disease (246/1614, 15.2%). Some participants were tested both in the acute and in the chronic phase (15/1614, 0.9%). A large number of individuals suffering from primary progressive aphasia (582/2256, 25.8%) were also evaluated. Impaired thematic role assignment, morphosyntactic processing, or both was also reported in 42 patients (1.9%) with glioma (II-IV grade tumors), as well as in 18 patients (0.8%) evaluated following left temporal lobectomy for intractable epilepsy.

To sum up, in the papers selected for the present review, comprehension was investigated more frequently than production. Thematic role assignment was investigated almost only through comprehension tasks that in most cases relied on sentence-picture matching or sentence-picture verification paradigms. In contrast, morphosyntactic impairment was assessed essentially only via speech production tasks, by means of picture description, storytelling, semi-structured interviews, etc. Studies were conducted most frequently in native speakers of English. Sentence processing difficulties were investigated in participants with stroke and neurodegenerative diseases, much less frequently with other pathologies. We will return to the possible implications of these dimensions in the “Discussion” section.

### Lesion-Mapping of Morphosyntactic and Thematic Role Deficits

Irrespective of whether they reported the coordinates of the correlation effects,^1^ studies exploiting voxel-based lesion-symptom correlations consistently showed the involvement of a left fronto-temporoparietal network in sentence processing.

#### Thematic Role Assignment

Most of the twenty-five experiments documenting selectively impaired thematic role assignment (Table 3) evidenced brain damage involving temporoparietal regions. The structures involved most frequently were the superior temporal gyrus (in 19/25 experiments, or 76%), the middle temporal gyrus (in 17/25, or 68%), the angular gyrus (in 11/25, 44%), and the supramarginal gyrus (10/25, or 40%). Less consistently, poor thematic role assignment correlated with the anterior temporal regions (3/25, 12%), the Heschl’s gyrus, and the planum temporale (1/25 experiment each, 4%). Prefrontal damage was reported in far fewer experiments than in temporoparietal structures. The pars opercularis and the pars triangularis of the inferior frontal gyrus were involved in 7/25 (28%) and in 6/25 (24%) experiments, respectively.

#### Morphosyntactic Processing

In the fifteen experiments selectively dealing with damaged morphosyntactic processing (Table 3), the emerging anatomical profile differed from that observed in the experiments focused on poor thematic role mapping. Lesions were observed in the left pars opercularis in 8/15 experiments (53.3%) and in the pars triangularis in 9/15 (60%). Parietotemporal damage affected the supramarginal gyrus, as shown in the 6/15 experiments (40%), and part of the angular gyrus, as shown in 2/15 (13.3%). Temporal lobe involvement was not as frequent. Lesions involved the middle temporal gyrus (4/15 experiments, or 26.7%), the superior temporal gyrus (3/15, or 20%), the anterior temporal area (2/15, or 13.3%), and the planum temporale (1/15, or 6.7%).

#### Thematic Role Assignment/Morphosyntactic Processing

In the 10 experiments dealing with both thematic role assignment and morphosyntactic processing (Table 3), prefrontal damage was observed in the pars opercularis (7/10, 70%) in the pars triangularis and pars orbitalis (6/10 each, 60%) and, less frequently, in the middle frontal gyrus (3/10, 30%). Temporoparietal damage was observed in the superior temporal gyrus (5/10, 50%), in the supramarginal gyrus (4/ 10, 40%), and in the middle temporal gyrus and angular gyrus (3/10 in both cases, 30%).

#### Sentence Comprehension Versus Production

Experiments on comprehension documented lesion-symptom correlations in the temporoparietal regions more frequently than in the prefrontal regions. The middle temporal gyrus was damaged in the 18/27 experiments (66.7%) and the superior temporal gyrus in 20/27 (74.1%). Lesions were observed also in the supramarginal gyrus and angular gyrus (11/27 experiments in both cases, 40.7%).

In experiments on production**,** prefrontal areas were involved somewhat more frequently than temporal regions. Poor performance was associated with lesions in the pars opercularis and the pars triangularis (which were sometimes damaged to a different degree — see, e.g., Matchin et al., 2020 vs Rogalski et al., 2011) in 11/20 experiments (55%) and in the middle frontal gyrus in 8/20 experiments (40%). The supramarginal gyrus was affected in 8/20 experiments (40%).

In the three experiments focusing on both modalities, poor performance was associated with damage to the pars opercularis and in the pars triangularis (2/3 experiments, or 66.6%), to the pars orbitalis (1/3 experiments, or 33.3%), to the middle temporal gyrus (1/3 experiments, or 33.3%), the superior temporal gyrus (2/3 experiments, or 66.6%), and the supramarginal gyrus and angular gyrus (1/3 experiments, or 33.3%).

## Meta-analysis Results

Twenty-seven publications met the criteria for inclusion in the meta-analysis and were considered relevant vis à vis thematic role assignment and morphosyntactic processing (Tables 2, 3, and 4). These publications covered a total of 1298 participants, 878 of which were patients with stroke (67.64%), 381 with brain atrophy (29.35%), 18 with lobectomy (1.39%), and 21 with tumor (1.62%).

**Fig. 2:**
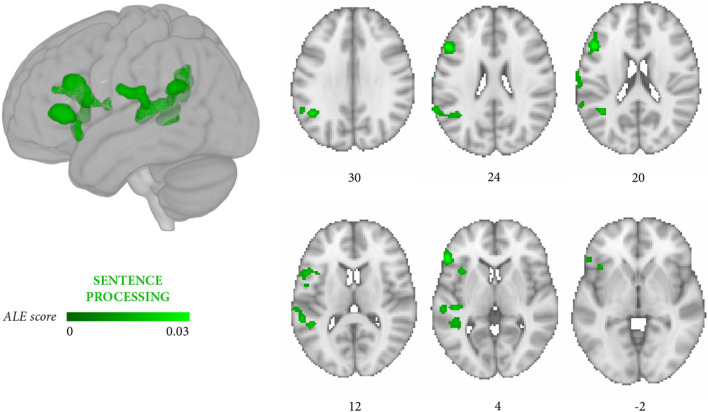
Meta-Analysis 1: clusters of convergence across studies focusing on thematic role assignment, on morphosyntactic processing abilities, or on both language processes (28 independent experiments, 249 foci, 1298 persons with aphasia). The significant clusters (cluster-level *p* < 0.05 FWE; cluster-forming threshold at voxel-level *p* < 0.001) are rendered on the standard MNI152 anatomical template. The color bar reflects activation likelihood estimation scores

The first meta-analysis included the data from 28 independent experiments (249 foci, 1298 persons with aphasia, Table 5). Its aim was to identify the areas whose damage was associated with poor processing of thematic role assignment, morphosyntactic processing, or both. Three significant left-hemisphere clusters emerged (*p* < 0.05 FWE), involving the inferior frontal gyrus, middle frontal gyrus, the insula, the precentral and postcentral gyri, the middle temporal gyrus, superior temporal gyrus, and supramarginal gyrus (Fig. 2, Table 6A).

**Table 6:** Meta-analysis clusters yielded by the revised activation likelihood estimation (ALE) algorithm implemented into GingerALE software (version 3.0.2; https://brainmap.org/ale/)

Cluster	Peak			Volume (voxels)	*Z* score	Anatomical structures	% of cluster
	*x* (mm)	*y *(mm)	*z*(mm)				
A. Meta-analysis 1. All experiments on TR and/or MS							
1	− 44	− 54	30	868	4.76	*Superior temporal gyrus*	51.5%
						*Supramarginal gyrus*	11.6%
						*Middle temporal gyrus*	10.8%
						*Postcentral gyrus*	8.9%
2	− 50	22	22	496	5.38	*Inferior frontal gyrus*	30.2%
						*Middle frontal gyrus*	24.2%
						*Insula*	18.8%
						*Precentral gyrus*	7.5%
3	− 52	28	2	171	5.33	*Inferior frontal gyrus*	98.8%
B. Meta-analysis 2 (exploratory). All experiments on TR							
1	− 48	22	20	182	4.68	*Inferior frontal gyrus*	36.3%
						*Middle frontal gyrus*	17%
						*Insula*	8.8%
2	− 56	− 40	10	107	3.71	*Middle temporal gyrus*	52.3%
						*Superior temporal gyrus*	23.4%
3	− 50	− 10	4	104	3.81	*Superior temporal gyrus*	41.3%
						*Precentral gyrus*	41.3%
						*Insula*	10.6%
4	− 52	32	6	103	4.08	*Inferior frontal gyrus*	99%
5	− 36	8	− 10	86	4.42	*Insula*	32.6%
						*Claustrum*	15.1%
6	− 58	− 28	6	78	4.55	*Superior temporal gyrus*	89.7%
						*Middle temporal gyrus*	10.3%
7	− 38	− 54	20	78	3.89	*Superior temporal gyrus*	55.1%
						*Middle temporal gyrus*	15.4%
						*Supramarginal gyrus*	15.4%
8	− 38	− 24	6	77	3.9	*Superior temporal gyrus*	41.6%
						*Insula*	27.3%
C. Meta-analysis 3 (exploratory). All experiments on MS							
1	-52	28		689	5.45	Inferior frontal gyrus	44.7%
						Middle frontal gyrus	18.7%
						Insula	17%
						Precentral gyrus	6.4%
2	-58	-38		146	4.53	Superior temporal gyrus	79.5%
						Postcentral gyrus	15.1%
D. Meta-analysis 4 (exploratory). Conjunction of all experiments on TR and MS							
*No significant clusters found*							
E. Meta-analysis 5 (exploratory). Direct comparison TR–MS							
1	− 46	− 16	− 8	12	2.33	*Superior temporal gyrus*	91.7%
2	− 44	− 22	− 2	2	1.73	*Insula*	100%
3	− 56	− 46	6	1	1.75	*Middle temporal gyrus*	100%
F. Meta-analysis 6 (exploratory). Direct comparison MS–TR							
	-48	38	20	6	2.29	Middle frontal gyrus	100%
	-44	32	18	1	2.07	Inferior frontal gyrus	100%

For each cluster, we specify the percentage involving the associated brain regions. The coordinates are in the MNI152 anatomical space. All clusters were located in the left hemisphere. A–C, significance threshold *p* < 0.05 FWE (based on 1000 permutations). D, significance threshold *p* < 0.05 FDR (based on 1000 permutations). E–F, *p* < 0.05 uncorrected

*MS* morphosyntactic processing, *TR* thematic role assignment

**Fig. 3:**
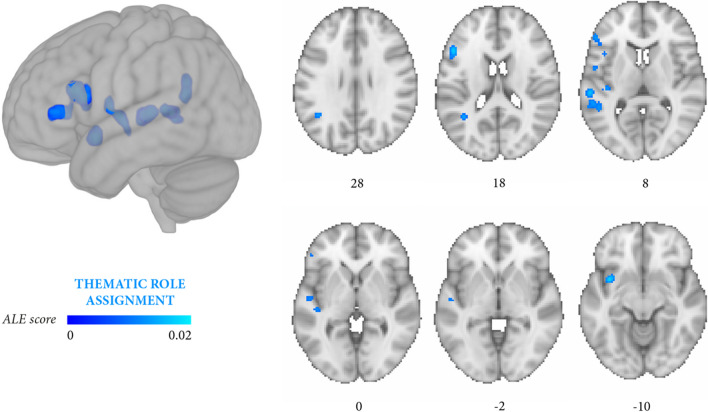
Meta-analysis 2 (exploratory): all experiments on thematic role assignment (19 independent experiments, 159 foci, 894 persons with aphasia). The significant clusters (cluster-level *p* < 0.05 FWE; cluster-forming threshold at voxel-level: *p* < 0.001) are rendered on the standard MNI152 anatomical template. The color bar reflects activation likelihood estimation scores

From the 19 independent experiments (159 foci, 894 persons with aphasia) that focused on thematic role assignment (experiments focusing on both thematic role assignment and morphosyntactic processing were also included, Table 5), the second meta-analysis retrieved eight significant clusters (*p* < 0.05 FWE), covering the left inferior frontal gyrus, middle frontal gyrus, precentral gyrus, insula, claustrum, middle temporal gyrus, superior temporal gyrus, supramarginal gyrus (Fig. 3, Table 6B).

**Fig. 4:**
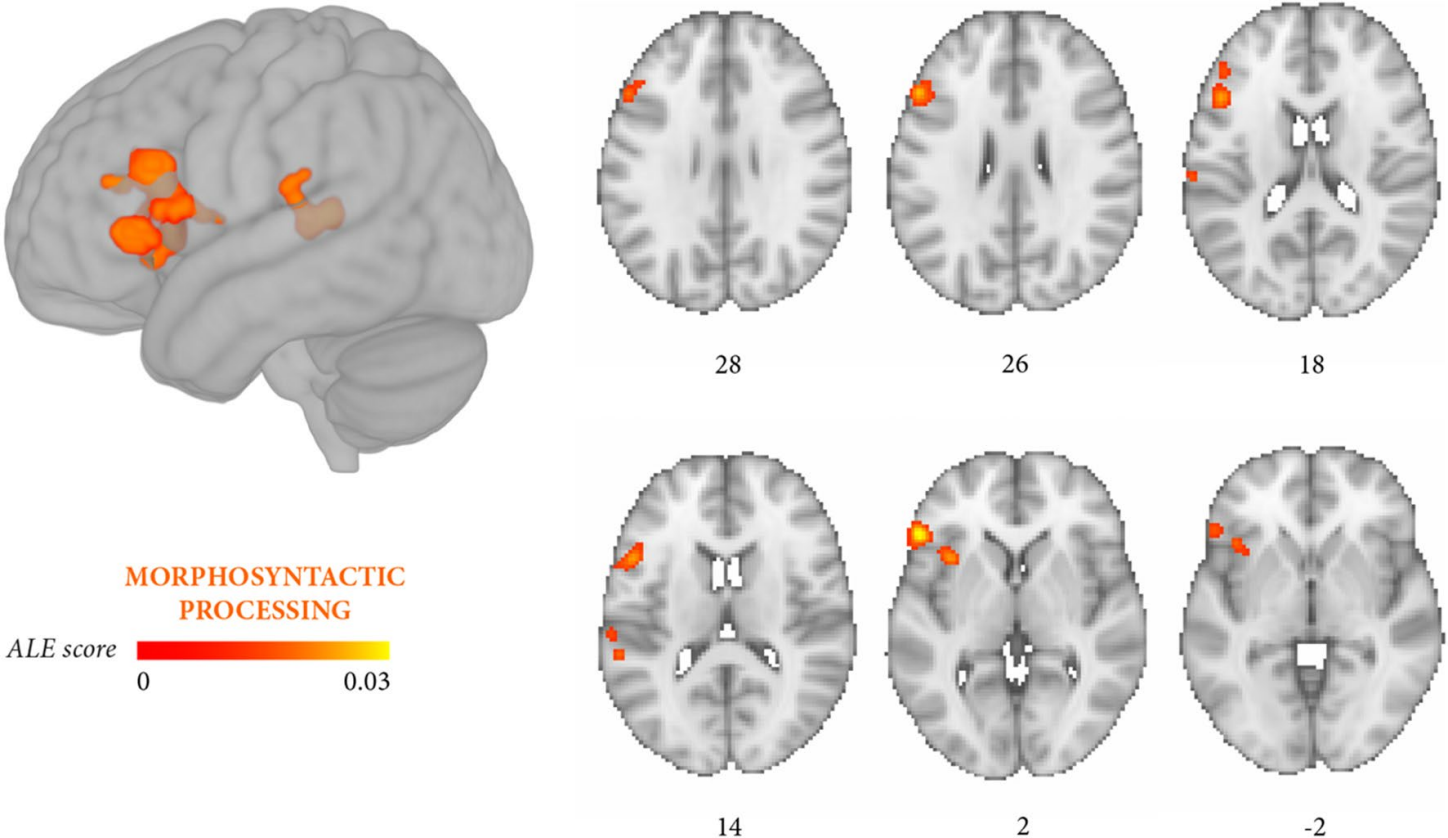
Meta-analysis 3 (exploratory): all experiments on morphosyntactic processing (16 independent experiments, 147 foci, 687 persons with aphasia). The significant clusters (cluster-level *p* < 0.05 FWE; cluster-forming threshold at voxel-level *p* < 0.001) are rendered on the standard MNI152 anatomical template. The color bar reflects activation likelihood estimation scores

The third meta-analysis included a total of 16 independent experiments (147 foci, 687 persons with aphasia) tapping morphosyntactic processing (experiments focusing on both thematic role assignment and morphosyntactic processing were also included, Table 5). Two significant clusters emerged (*p* < 0.05 FWE), involving the left inferior frontal gyrus, middle frontal gyrus, the insula, the precentral and postcentral gyri, and the superior temporal gyrus (Fig. 4, Table 6C).

No significant clusters emerged from the conjunction analysis between experiments specifically focusing on thematic role assignment (14 independent experiments, 102 foci, 701 persons with aphasia, Table 5) and morphosyntactic processes (10 independent experiments, 90 foci, 466 persons with aphasia, Table 5) (*p* < 0.05 FDR, Table 6D), even when the significance threshold was lowered to *p* < 0.05 uncorrected.

**Fig. 5:**
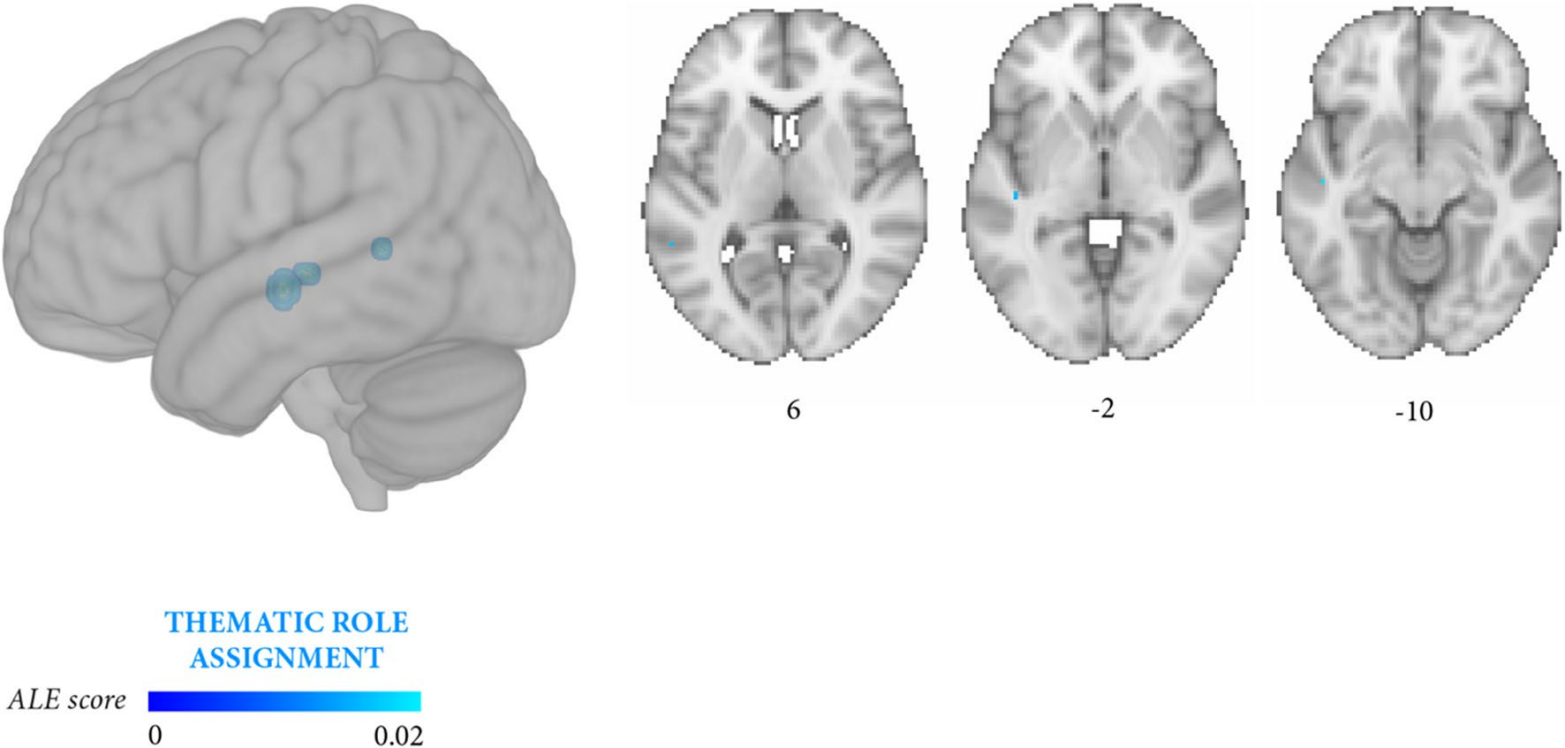
Meta-analysis 5 (exploratory): stronger convergence likelihood for thematic role assignment (14 independent experiments, 102 foci, 701 persons with aphasia) than for morphosyntactic processing (10 independent experiments, 90 foci, 466 persons with aphasia). Likelihood clusters (cluster-level *p* < 0.05 uncorrected) are rendered on the standard MNI152 anatomical template. The color bar reflects activation likelihood estimation scores

**Fig. 6:**
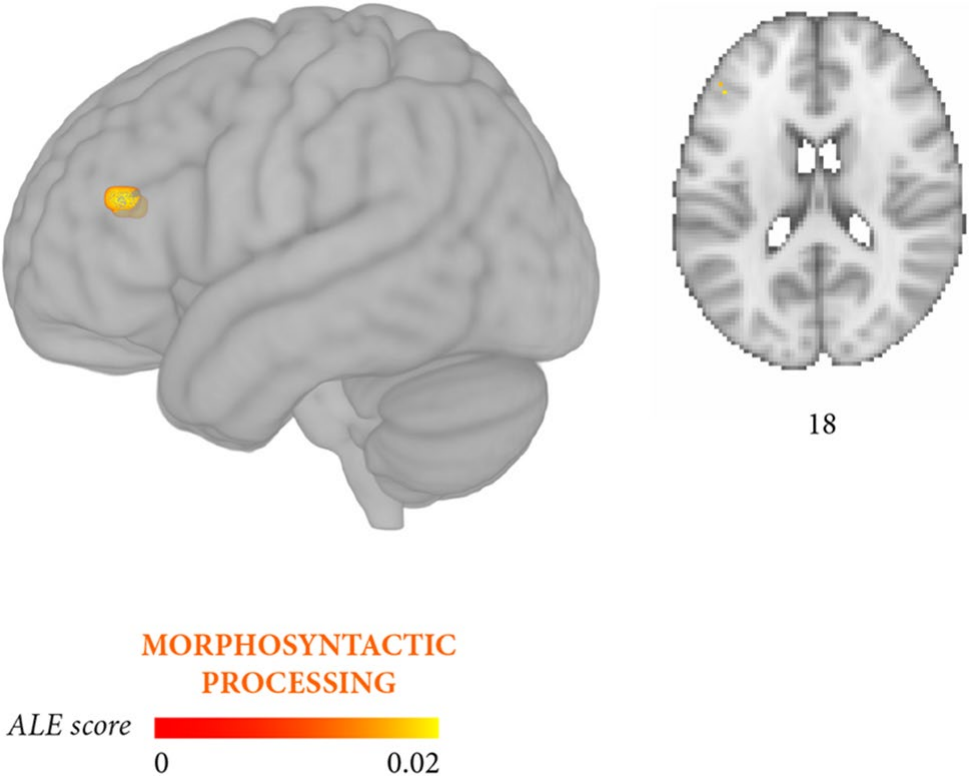
Meta-analysis 6 (exploratory): stronger convergence likelihood for morphosyntactic processing (10 independent experiments, 90 foci, 466 persons with aphasia) than for thematic role assignment (14 independent experiments, 102 foci, 701 persons with aphasia). Likelihood clusters (cluster-level *p* < 0.05 uncorrected) are rendered on the standard MNI152 anatomical template. The color bar reflects activation likelihood estimation scores

The contrasts between the experiments that specifically focused on thematic role assignment vs morphosyntactic processing did not yield significant clusters in either direction (*p* < 0.05 FDR). Lowering the significance threshold to *p* < 0.05 uncorrected for exploratory purposes yielded clusters correlated to thematic role assignment more than morphosyntactic processing in the left middle temporal gyrus, superior temporal gyrus, and the insula (Fig. 5, Table 6E), and clusters correlated to morphosyntactic processing more than to thematic role assignment in the left inferior frontal gyrus and middle frontal gyrus (Fig. 6, Table 6F).

## Discussion

Disorders of sentence processing are among the most frequently investigated language deficits in aphasia. In recent years, several studies set out to identify the brain regions whose damage leads to difficulties in sentence comprehension and/or production. For the present project, a systematic search of the literature on persons with aphasia and a coordinate-based meta-analysis of lesion-symptom mapping studies was carried out to investigate the neurofunctional correlates of impaired thematic and morphosyntactic processing. Behavioral and neuroimaging data of 2256 persons with aphasia were selected and reviewed. Data from 1298 participants, for whom whole-brain analysis and coordinates were available, were used to estimate the anatomical likelihood of the language processes under exam. A total of 43 papers were retained for review. Of these, 27 met the prerequisites for inclusion in the meta-analysis.

The meta-analysis included all 27 papers on impaired sentence processing (irrespective of whether they focused on thematic role assignment, morphosyntactic processing, or both). It revealed a large network of converging areas of brain damage (Fig. 2). Poor sentence processing correlated with prefrontal damage involving the inferior frontal gyrus, middle frontal gyrus, and the insula and with temporal and temporoparietal damage involving the superior temporal gyrus, middle temporal gyrus, and supramarginal gyrus (Table 6A). This result is comparable with that of studies that considered both processes together (e.g., Faroqi-Shah et al., 2014, Henry, 2009; Kamminga et al., 2016). The small number of eligible papers prevented statistically powered fractionation of the 27 papers in specific meta-analysis subsets on, respectively, thematic role assignment or morphosyntactic processing. However, given the current lack of meta-analytic evidence on the specific lesion-symptom correlates of thematic role and morphosyntactic processing and the urgent need — as mentioned in the “Introduction” section, and as addressed in further detail below — to refocus priorities in future experiments on these topics, we believe that preliminary results are worth reporting, although they require particular consideration due to their exploratory nature.

The two meta-analyses focusing more specifically on thematic roles and on morphosyntactic errors yielded partially distinguishable outcomes. Lesions associated with thematic role impairment converged in a large temporal cluster (middle temporal gyrus, superior temporal gyrus) and in a smaller prefrontal cluster (inferior frontal gyrus, middle frontal gyrus, insula) (Fig. 3, Table 6B), whereas the reverse damage profile applied to morphosyntactic processing (Fig. 4, Table 6C). In this latter case, lesions converged in several prefrontal clusters distributed along the inferior frontal gyrus and middle frontal gyrus and, less extensively, in temporal regions (middle temporal gyrus, superior temporal gyrus). Directly contrasting thematic role assignment and morphosyntactic processing (Fig. 5, Table 6E) highlighted three small thematic role-related clusters in the superior temporal gyrus, middle temporal gyrus, and the posterior portion of the insula, whereas the reverse contrast (Fig. 6, Table 6F) retrieved two small, morphosyntactic-related clusters in the inferior frontal gyrus and middle frontal gyrus. Although it remains quite possible that the two processes share some underlying components, these exploratory results tentatively suggest that thematic role and morphosyntactic processes involve more extensively temporal/temporoparietal and prefrontal regions, respectively.

### Neurofunctional Correlates of Thematic Role Mapping

Both the literature review and the meta-analysis confirm the role of temporal and temporoparietal structures in the assignment of thematic roles. The superior temporal gyrus was involved in 19/25 experiments (76%). Its posterior portion was deemed critical by Amici et al. (2007); LaCroix et al. (2020); Lukic et al., (2017); Pillay et al. (2017); Riccardi et al. (2022); Rogalsky et al. (2018); Sheppard et al. (2022); Thothathiri et al. (2012); and Wu et al. (2007), whereas its anterior part was more relevant according to Dronkers et al. (2004); Charles et al., (2014), and Wu et al. (2007). Several studies assigned a role also to the posterior part of the middle temporal gyrus (Amici et al., 2007; Baldo & Dronkers, 2007; Dronkers et al., 2004; Kristinsson et al., 2020; Matchin et al., 2022; Riccardi et al., 2022; Thothathiri et al., 2012), whereas the anterior temporal region including the temporal pole emerged infrequently (Lukic et al., 2020; Kim et al., 2010; Magnusdottir et al., 2013). The critical role of left temporoparietal regions is supported also by the significant involvement of the angular gyrus and supramarginal gyrus (in 44% and 40% of the experiments, respectively, as well as in our meta-analysis). The role of left temporoparietal regions was also confirmed by ROI analyses (e.g., Newhart et al., 2012). These observations suggest that temporal and temporoparietal regions are critical for thematic role assignment, with temporal regions possibly playing the greater role. It cannot be ruled out that the extensive involvement of the superior temporal gyrus that emerged in relation to thematic role assignment is in part task-specific, as sentence comprehension was investigated almost exclusively by auditory comprehension tasks, in which early stimulus processing involves the auditory radiations running deep to the posterior insula and the primary and non-primary auditory cortices in the temporal lobe (for reviews, see Pickles, 2015). Furthermore, it appears that the differences at the brain level cannot be readily explained by methodological differences concerning the use of different tasks or different linguistic structures. For example, studies that find, e.g., anterior (Charles et al., 2014; Dronkers et al., 2004; Wu et al., 2007) as opposed to posterior (e.g., Amici et al., 2007; LaCroix et al., 2020; Lukic et al., 2017) areas of the temporal lobe used a similar variety of behavioral measures (see Table 3).

Notably, some investigations focusing on thematic role mapping involved prefrontal regions (e.g., Kim et al., 2010; Peelle et al., 2008), as also evidenced by our thematic role-specific meta-analysis (Fig. 3, Table 6B). This result must be taken with some caution, as these studies contrasted thematic role assignment with measures of lexical-semantic difficulties but did not consider morphosyntactic impairments, which may influence thematic role assignment (e.g., Tyler et al., 2011; Wilson et al., 2016). In Kinno et al., (2009, 2014) prefrontal involvement results from a sampling bias, as only patients with frontal lobe gliomas were recruited. ROI analyses showed the involvement of the pars triangularis and opercularis in Kristinsson et al. (2020); LaCroix et al. (2020), and Sheppard et al. (2022). From our survey of the task features in these different studies (see Table 3), no clear differences emerged compared to other studies that did not identify frontal involvement in the lesion-symptom analyses, in addition to a prominent heterogeneity among all studies in etiology, the language used, and lesion location or area of analysis.

### Neurofunctional Correlates of Morphosyntactic Processing

In the set of reviewed studies (Ash et al., 2013, 2015; Borovsky et al., 2007; DeLeon et al., 2012; Grossman et al., 2013; Matchin et al., 2020, 2022; Mirman et al., 2019; den Ouden et al., 2019; Wilson et al., 2010a; Henseler et al., 2014; Matchin et al., 2021; Rogalski et al., 2011; Sapolsky et al., 2010), morphosyntactic impairment correlated with damage to left prefrontal, parietal and, less consistently, temporal regions. In the meta-analysis including all the 16 studies that considered morphosyntactic processes (either exclusively or in association with thematic role assignment), the inferior frontal gyrus and the middle frontal gyrus were involved more extensively than temporal and parietal regions. This result is consistent with the direct contrast meta-analysis between experiments on morphosyntactic processing versus thematic role assignment, that also yielded the inferior and middle frontal gyri. Correlations with the posterior two-thirds of the inferior frontal gyrus emerged in 11/15 (73.3%) experiments focused on morphosyntactic processing and in 7/10 (70%) investigations dealing with both morphosyntactic processing and thematic role assignment. Prefrontal involvement is confirmed by ROI-based analyses in Mesulam et al. (2021) and Wilson et al. (2011). Our coordinate-based meta-analysis on morphosyntactic processes also involved the superior temporal gyrus (Fig. 4, Table 6C).

### The Neurofunctional Correlates of Sentence Processing

**Fig. 7:**
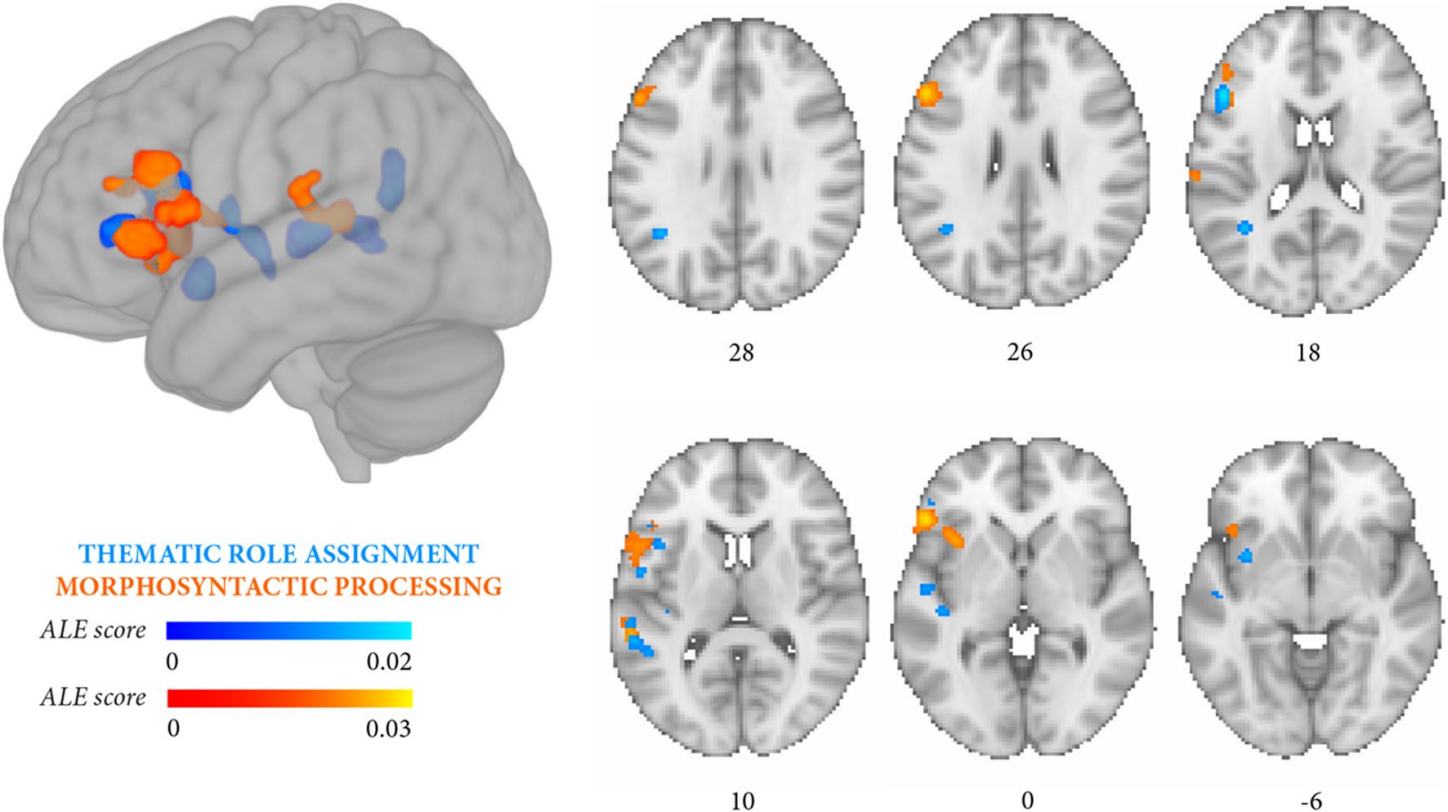
Overlap of the clusters shown in Fig. 3 (thematic role assignment) and Fig. 4 (morphosyntactic processes). Note that thematic and morphosyntactic processes correlate with both overlapping and distinct clusters in the prefrontal regions (inferior frontal gyrus, middle frontal gyrus, anterior insula) and temporal/temporoparietal regions (superior temporal gyrus)

Turning back to the consideration of sentence processing as a whole (43 papers in our systematic review, 27 of which were included in meta-analysis 1), our survey indicated that, at least as regards thematic role assignment and morphosyntactic processes, sentence comprehension and production correlate with an extensive fronto-temporoparietal network. On the other hand, visual inspection of Figs. 3 and 4 (see Fig. 7 for a direct overlap) suggests that poor thematic role assignment involves damage to temporal and temporoparietal regions (middle temporal gyrus, superior temporal gyrus, supramarginal gyrus) more than to prefrontal areas and that, conversely, morphosyntactic difficulties are associated with damage to inferior frontal gyrus and middle frontal gyrus more than with temporal and temporoparietal lesions. The results for thematic difficulties confirm recent studies showing a strong correlation with temporal and temporoparietal damage (e.g., Magnusdottir et al., 2013; Rogalsky et al., 2018; Thothathiri et al., 2012). Furthermore, and at odds with the conclusions drawn in the same studies, the systematic literature review shows that morphosyntactic deficits and, to a lesser extent, thematic role difficulties also correlate with left prefrontal damage.

In light of these results, one could wonder if the apparent asymmetry is an artifact of the distribution of the reviewed literature addressing the two features. In fact, all the studies evaluating only thematic role assignment (*n* = 25) focused on comprehension, and all those investigating only morphosyntactic processing (*n* = 15) focused on production. The remaining 10 papers that addressed both thematic role assignment and morphosyntactic processing studied production (*n* = 5), comprehension (*n* = 2), or both (*n* = 3). This could generate a strong bias, especially considering that results reported in the “Sentence Comprehension Versus Production” section show that the regions retrieved from the analysis of studies on thematic role assignment and morphosyntactic processing are very similar to those retrieved from the analysis of studies on comprehension and production, respectively. At issue here, then, is establishing whether the distinction between language processes reported in the “Meta-analysis Results” section can be reduced to that between comprehension and production, as opposed to being an indication that the neural substrates for morphosyntactic and thematic processes are distinguishable.

An unambiguous conclusion is difficult, as both the comprehension/production and the thematic/morphosyntactic contrast involve the fronto-temporoparietal structures of the language network. From the modality perspective, neuroimaging studies in control participants have argued both for the separability (Giglio et al., 2022; Matchin et al., 2020) and for the overlap (Hu et al., 2023) of the neural substrates recruited by input and output language processes. Evidence from aphasia is consistent with the view that comprehension and production involve both shared and separate neural substrates (Matchin et al., 2020; Lukic et al., 2020) — a view supported by massive evidence of both co-occurring and selective disorders of specific aspects of language comprehension and production following left hemisphere damage. In principle, then, a modality-biased account cannot be ruled out completely. Overall, however, it remains very unlikely. First, the areas retrieved in each analysis extend well beyond the structures involved in articulation and hearing and include regions unanimously deemed as critical for sentence processing. Secondly, also when considered separately, thematic role and morphosyntactic processes were linked to both prefrontal and temporal areas. In other words, and against a strictly modality-biased interpretation, analyses of studies on morphosyntax (that focused on production) also retrieved temporal regions, and analyses of thematic processes (that focused on comprehension) also retrieved frontal regions. Evidence from agrammatic aphasia also militates against the modality account. For example, 38 participants with agrammatism damage to Broca’s area correlated with both thematic and morphosyntactic errors in sentence comprehension (e.g., Table 2 in Caramazza et al., 2005). A modality-based account cannot accommodate this behavioral profile, as prefrontal damage should affect production, not comprehension. Thus, even though the modality bias cannot be completely ruled out nor quantified, the distinguishable outcomes of our analyses of thematic and morphosyntactic processes suggest a genuine distinction between language mechanisms.

Inspection of Fig. 7 shows that the disruption of morphosyntactic and thematic role processes correlates with damage to both overlapping and distinctive regions (see for example Lukic et al., 2020). There is an overlap in a prefrontal region in the inferior frontal gyrus–middle frontal gyrus and in a temporal region in the superior temporal gyrus. In processing reversible sentences, these structures could be involved in the necessary integration of thematic and morphosyntactic features. In production, thematic role assignment must be integrated with morphosyntactic features such as determiner-noun-adjective agreement, subject-verb agreement, and case, person, and tense markings. In comprehension, correct processing of morphosyntactic features is needed to disambiguate sentence structure for thematic role assignment (e.g., in passive sentences). While each of the two processing steps may be influenced by dimensions that do not affect the other and may be implemented in distinct neural substrates, the two must interact closely. Further research will have to establish whether the overlapping prefrontal and temporal regions are equally critical for both processes, at all stages of sentence processing, and in both comprehension and production.

The neural network emerging from the present study is also in line with meta-analyses of studies on language processes in neurotypical subjects (Bulut, 2022; Stefaniak et al., 2021; Walenski et al., 2019, Vigneau et al., 2011; Price, 2012). Increased BOLD activity during comprehension emerged in the posterior temporal areas and in part of the frontal areas (Stefaniak et al., 2021). The same areas were retrieved from the comparison between comprehension and production in the meta-analysis by Walenski et al. (2019) that showed the involvement of large portions of the temporal lobe (anterior, posterior, and temporo-occipital), and a correlation with the inferior frontal gyrus and the inferior parietal cortex, including the angular gyrus. Impaired production correlated more strongly to frontal areas (Stefaniak et al., 2021) and especially the middle and the inferior frontal gyrus (Walenski et al., 2019). Conjunction analyses between sentence comprehension and production (Walenski et al., 2019) retrieved the middle frontal gyrus, the superior frontal gyrus, the supplementary motor area, and the posterior portion of the middle temporal gyrus. Vigneau et al. (2011) focused on frontal and temporal regions. They linked the pars opercularis to syntactic processing and the posterior part of the superior temporal gyrus to sentence and text processing. The left inferior frontal gyrus also emerged from a meta-analysis on inflectional morphology at the single-word processing level by Bulut (2022), who concluded that the inferior frontal gyrus provides the neural basis of morphology and inflectional syntax. Interestingly, meta-analyses of fMRI studies rely on a larger paper pool as compared to investigations based on structural MRI, like the present work or the one by Na et al. (2022). The latter work consisted of a meta-analysis on 25 lesion-symptom correlation studies in English speakers with post-stroke aphasia, considering language function in general, as well as specifically language comprehension, speech production, speech fluency, repetition, naming, reading, phonology, and semantics. When considering the full set of 25 articles, Na et al. (2022) found the involvement of a left fronto-temporoparietal network, in large agreement with our findings in meta-analysis 1. In the analysis restricted to language comprehension, they found convergent correlations in the left superior temporal gyrus and fusiform gyrus, whereas for production, the effects were in the left precentral gyrus, insula, and superior temporal gyrus. These results for comprehension and production overlap only in part with the dissociation we found for thematic role assignment (meta-analyses 2 and 5) versus morphosyntactic processing (meta-analyses 3 and 6), despite the fact that, as discussed above, the thematic role assignment-morphosyntactic processing dissociation in our selection of studies was largely confounded by the comprehension-production dichotomy. It is worth noting here that this divergence, which in the case of Na et al. (2022) is most likely due to the inclusion of studies focusing not only on sentence processing but also on a variety of tasks involving single words, further supports the view that our meta-analytic results for thematic role assignment versus morphosyntactic processing cannot be fully explained by the mere dissociation between comprehension and production, but rather reflect, at least in part, a genuine dissociation between the two processes considered here.

## Limitations

A thorough search of the literature on disorders of thematic role assignment and of morphosyntactic processing in persons with aphasia was conducted, to identify the neurofunctional correlates of these two aspects of sentence processing. The main limitation of the present report is that the number of eligible studies dealing with their neural correlates is surprisingly small, to the point that meta-analyses with sufficient statistical power were allowed only on the overall paper sample. All in all, the results are promising but pose several questions that concern particularly the risk of bias posed by the selected studies in relation to behavioral, neurofunctional, and methodological issues.

As regards behavioral dimensions, caution is suggested by the variability of the experimental paradigms (Table 3). Across studies, comprehension was mostly assessed by presenting a reversible sentence and asking the participant to show comprehension by selecting the correct response from thematic alternatives. The diversity of means used to reach this goal does not permit an unequivocal interpretation of the outcomes. A significant drawback is that damage to thematic role assignment was evaluated by considering the overall performance in tasks including small numbers of syntactically heterogeneous stimuli that varied greatly in complexity, ranging from simple declaratives to subject- and object-relatives, subject- and object-clefts, and Wh-questions. All these sentences do require thematic role assignment, but they recruit additional cognitive and linguistic skills to a variable but significant extent. Furthermore, administering small numbers of syntactically heterogeneous sentences may not license strong conclusions on specific sentence types. Another critical issue is that understanding of reversible sentences was assessed via different tasks, such as sentence-picture matching, sentence-picture verification, and meaning acceptability, that pose different demands regarding the ease of the decision (e.g., depending on the number of alternative responses from which the participant must choose explicitly or implicitly). Both considerations also apply to studies on production, in which an even greater variety of tasks and expected responses was exploited, including picture description, sentence completion, primed sentence production, sentence anagram, semi-structured interviews, and story (re)telling.

A further and very serious limitation is that in most cases attention was focused exclusively on thematic analysis, and target sentences were paired only with role reversal foils. However, as stated in the “Introduction” section, morphosyntactic analysis is just as indispensable as thematic analysis in both comprehension and production, even in morphologically poor languages like English. Since morphosyntactic foils were not included in the experimental tasks, errors interpreted as being the consequence of thematic deficits might have been determined by co-occurring but neglected morphosyntactic difficulties. In comprehension, for example, failure to process the *by*-phrase in a passive sentence may result in incorrectly assigning the agent role to the first argument instead of the second, because of the “first noun bias.” In the absence of some independent measure of morphosyntactic processing abilities, this error would be scored as thematic. Consequently, the neurofunctional correlates of thematic analysis proposed on these bases must be taken with caution. Even though results show that comprehending reversible sentences involves a fronto-temporoparietal network, if and to what extent each component of this network is involved in thematic analysis, in morphosyntactic processes, or in both remains to be established more clearly.

These limitations must be addressed in future studies. Different experimental paradigms will always be used, of course, but better control should be exerted when designing experimental tools. To account more straightforwardly for deviant performance, tasks should include more stimuli but of fewer types, lest the mechanisms yielding incorrect responses be inextricable. Complex syntactic structures should be probed, but only in association with adequate measures of the additional cognitive resources needed to process them (e.g., short-term memory). In addition, whether focusing on comprehension or production, experimental tasks should assess both morphosyntactic and thematic processes, possibly in identical sentence contexts. Studies in languages morphologically richer than English can provide a more comprehensive picture of the interplay between thematic and morphosyntactic processes.

From the neural viewpoint, the heterogeneity of brain damage in the sampled population poses obvious problems. Most persons with aphasia in reported studies suffered from cerebrovascular or neurodegenerative disease, and some papers reported on individuals with brain gliomas and epilepsy. These conditions differ along relevant clinical dimensions, such as onset modality (abrupt vs slow), disease course (limited but possible recovery vs inexorable worsening), lesion type (tissue disruption vs infiltration), and damage distribution (superficial and deep unilateral lesions constrained by the vascular tree vs asymmetric but often bilateral damage constrained by network connections but not by blood vessel distribution). These factors may significantly influence neurofunctional correlations. Diseases with sudden as opposed to gradual onset (e.g., stroke vs tumor and neurodegeneration) give different opportunities for compensatory functional reorganization. Consequently, the behavioral deficit documented in slowly evolving lesions may not be transparently related to the original function of the affected region(s). Different conditions also pose specific challenges in lesion reconstruction. Necrotic tissue is easily identifiable by structural MRI images in most stroke cases, but in the face of slowly evolving gliomas or atrophy, the boundary of the lesion is not always clear, and damage may be partial. In recent years, it has been recommended that for the purpose of accurate localization, lesion volume be controlled in lesion-symptom correlation analyses (de Marco & Turkeltaub, 2018; Moore et al., 2024). This is not the case in many available reports (Table 4), but future investigations should comply with this advice. Accumulating an increasing number of studies will permit us to consider the effects that different etiologies may exert on the behavioral and the neural correlates of sentence comprehension.

Finally, some studies retained for the analyses reported in this manuscript used partially overlapping samples — not an uncommon problem in ALE-based meta-analyses (Müller et al., 2018). An unspecified number of participants overlap with Thothathiri et al. (2012) and Mirman et al. (2019), and 21 participants overlap with den Ouden et al. (2019) and Matchin et al. (2020). An even more inextricable situation affects Matchin et al. (2022). This study involves two samples of persons with aphasia: one performed a non-canonical sentence comprehension task (Group 1) and the other a speech production task (Group 2). Group 1 includes 47 participants from den Ouden et al. (2019) and 26 from both den Ouden et al. (2019) and Kristinsson et al. (2020). Group 2 includes 39 participants from den Ouden et al. (2019), 21 from both den Ouden et al. (2019) and Kristinsson et al. (2020), and 32 from Matchin et al. (2020). Finally, Group 1 and Group 2 overlap for an unspecified number of participants. However compelling individually, these studies create serious problems for review and meta-analyses of neuroimaging studies, as it becomes virtually impossible to properly account for statistical dependence between individual observations. The effect size multiplicity issue in the context of meta-analyses of neuroimaging studies has been an especially challenging problem to address (Müller et al., 2018). Available methods that are amenable to low-dimensional datasets (e.g., behavioral or clinical treatment effect sizes), such as the multivariate or three-level meta-analytic approaches (Cheung, 2019), are not readily applicable to coordinate-based neuroimaging meta-analyses. As pointed out by an anonymous reviewer, adequately handling statistical dependencies between neuroimaging datasets would require a not-yet-available multivariate random-effects approach that can synthesize the spatial dependencies among coordinates. Notwithstanding, in order to at least partially remediate the methodological drawback in counting studies with partially overlapping samples, we performed a series of additional controls on our data. First, we carefully inspected the resulting output produced by the GingerALE software. This output provides information on the contribution of each individual study to the significant meta-analytic clusters, thus allowing us to estimate the likely impact of excluding one or more studies from the meta-analysis. Second, we sought confirmation for these estimates through a sensitivity analysis approach, in which we repeated every meta-analysis after the exclusion of combinations of studies yielding sample overlaps, including the least favorable combination as projected by GingerALE (Supplementary Table 1 and Supplementary Text). Importantly, the sensitivity analyses confirmed distinguishable associations between morphosyntactic processing and prefrontal areas and between thematic role assignment and temporoparietal areas. Thus, reassuringly, in our case, the effect size multiplicity bias does not seem to substantially modify the distinguishable outcomes of the analyses focused on thematic role assignment and on morphosyntactic processing.

How to overcome the sample overlap bias in the future remains unclear. Ideally, behavioral and neuroimaging documentation for individual participants should be made available on shared repositories.

## Conclusions

The processing of semantically reversible sentences involves a neural network that includes prefrontal areas (inferior frontal gyrus, middle frontal gyrus, insula) and temporal areas (superior temporal gyrus, but also middle temporal gyrus) and extends into supramarginal gyrus and angular gyrus. Thematic role assignment correlates mainly with the posterior part of the superior and middle temporal gyrus, extending to the inferior part of the supramarginal and angular gyri and involving the inferior frontal gyrus, albeit to a lesser extent. In contrast, morphosyntactic processing correlates mainly with the inferior and middle frontal gyri and, to a lesser extent, with temporal and temporoparietal regions. Exploratory meta-analyses contrasting thematic role assignment and morphosyntactic processing suggest a stronger association of the inferior and middle frontal gyrus with morphosyntactic processing than thematic role processing and the reverse correlation pattern for the middle temporal gyrus. These results confirm the correlation of temporal and temporoparietal regions with thematic role processing, documented in several recent studies. At variance with these investigations, however, data strongly argue for the involvement of prefrontal structures in sentence processing. Future research should focus on defining the role of each area and on the dynamics of the interactions in the network.

Results must be taken with caution. The available literature is limited (43 papers eligible for the literature review, 27 for the meta-analysis). Most studies of thematic role mapping disorders focus on sentence comprehension and most investigations on morphosyntactic difficulties in sentence production. Participants, who were for the most part native speakers of English, were tested with very different tasks and were affected by heterogeneous neurological conditions that differently affect linguistic phenotype and ease of damage compensation, thus yielding potentially problematic lesion-symptom mapping results.

To overcome these limitations, future studies should increase the database. Working with larger numbers will not suffice to solve outstanding issues, however. Including assessments of morphosyntactic and thematic processes in both comprehension and production and possibly extending analyses to languages with richer morphosyntax than English will be important. Ideally, investigations should focus on participants with homogeneous etiologies and evaluated with stimuli of comparable thematic and morphosyntactic difficulty. Ways to control for participant sample overlaps should be articulated. Consideration of these characteristics in future studies will allow us to more clearly understand the neurofunctional correlates of the two linguistic processes analyzed in this manuscript and to identify the substrates involved in the production and in the comprehension of sentences.

## Supplementary Information

Below is the link to the electronic supplementary material.

Supplementary file1 (DOCX 9 KB)

Supplementary file2 (XLSX 872 KB)

## Data Availability

Data are available from the corresponding author upon request.

## Footnotes

1 In case they did not, the studies were excluded from our coordinate-based meta-analysis (Table 4).

## References

[CR1] Amici, S., Brambati, S. M., Wilkins, D. P., Ogar, J., Dronkers, N. L., Miller, B. L., & Gorno-Tempini, M. L. (2007). Anatomical correlates of sentence comprehension and verbal working memory in neurodegenerative disease. *Journal of Neuroscience,**27*(23), 6282–6290. 10.1523/JNEUROSCI.1331-07.2007 17554002 10.1523/JNEUROSCI.1331-07.2007 PMC6672164

[CR2] Ash, S., Evans, E., O’Shea, J., Powers, J., Boller, A., Weinberg, D., Haley, J., McMillan, C., Irwin, D. J., Rascovsky, K., & Grossman, M. (2013). Differentiating primary progressive aphasias in a brief sample of connected speech. *Neurology,**81*(4), 329–336. 10.1212/WNL.0b013e31829c5d0e 23794681 10.1212/WNL.0b013e31829c5d0e PMC3772830

[CR3] Ash, S., Olm, C., McMillan, C. T., Boller, A., Irwin, D. J., McCluskey, L., Elman, L., & Grossman, M. (2015). Deficits in sentence expression in amyotrophic lateral sclerosis. *Amyotrophic Lateral Sclerosis & Frontotemporal Degeneration,**16*(1–2), 31–39. 10.3109/21678421.2014.974617 25482157 10.3109/21678421.2014.974617 PMC4372458

[CR4] Aziz, M. A. A., Hassan, M., Razak, R. A., & Garraffa, M. (2020). Syntactic abilities in Malay adult speakers with aphasia: A study on passive sentences and argument structures. *Aphasiology,**34*(7), 886–904. 10.1080/02687038.2020.1742283

[CR5] Baldo, J. V., & Dronkers, N. F. (2006). The role of inferior parietal and inferior frontal cortex in working memory. *Neuropsychology,**20*(5), 529. 10.1037/0894-4105.20.5.529 16938015 10.1037/0894-4105.20.5.529

[CR6] Baldo, J. V., & Dronkers, N. F. (2007). Neural correlates of arithmetic and language comprehension: A common substrate? *Neuropsychologia,**45*(2), 229–235. 10.1016/j.neuropsychologia.2006.07.014 16997333 10.1016/j.neuropsychologia.2006.07.014

[CR7] Bastiaanse, R. (1995). Broca’s Aphasia: A syntactic and/or a morphological disorder? *A Case Study. Brain and Language,**48*(1), 1–32. 10.1006/brln.1995.1001 7712146 10.1006/brln.1995.1001

[CR8] Bates, E. A., Friederici, A. D., Wulfeck, B. B., & Juarez, L. A. (1988). On the preservation of word order in aphasia: Cross-linguistic evidence. *Brain and Language,**33*(2), 323–364. 10.1016/0093-934X(88)90072-7 3359173 10.1016/0093-934x(88)90072-7

[CR9] Berndt, R. S., Mitchum, C. C., & Haendiges, A. N. (1996). Comprehension of reversible sentences in “agrammatism”: A meta-analysis. *Cognition,**58*(3), 289–308. 10.1016/0010-0277(95)00682-6 8871341 10.1016/0010-0277(95)00682-6

[CR10] Bishop, D. V. M. (1989). *Test for the reception of grammar* (2nd ed.). Medical Research Council.

[CR11] Bornkessel, I., Zysset, S., Friederici, A. D., Von Cramon, D. Y., & Schlesewsky, M. (2005). Who did what to whom? The neural basis of argument hierarchies during language comprehension. *NeuroImage,**26*(1), 221–233. 10.1016/j.neuroimage.2005.01.032 15862222 10.1016/j.neuroimage.2005.01.032

[CR12] Borovsky, A., Saygin, A. P., Bates, E., & Dronkers, N. (2007). Lesion correlates of conversational speech production deficits. *Neuropsychologia,**45*(11), 2525–2533. 10.1016/j.neuropsychologia.2007.03.023 17499317 10.1016/j.neuropsychologia.2007.03.023 PMC5610916

[CR13] Brookshire, R. H., & Nicholas, L. E. (1980). Verification of active and passive sentences by aphasic and nonaphasic subjects. *Journal of Speech, Language, and Hearing Research,**23*(4), 878–893. 10.1044/jshr.2304.878 10.1044/jshr.2304.878 7442218

[CR14] Bulut, T. (2022). Neural correlates of morphological processing: An activation likelihood estimation meta-analysis. *Cortex,**151*, 49–69. 10.1016/j.cortex.2022.02.010 35397379 10.1016/j.cortex.2022.02.010

[CR15] Canu, E., Agosta, F., Imperiale, F., Ferraro, P. M., Fontana, A., Magnani, G., Mesulam, M.-M., Thompson, C. K., Weintraub, S., Moro, A., Cappa, S. F., & Filippi, M. (2019). Northwestern Anagram Test-Italian (Nat-I) for primary progressive aphasia. *Cortex,**119*, 497–510. 10.1016/j.cortex.2019.08.007 31527011 10.1016/j.cortex.2019.08.007 PMC6785992

[CR16] Caplan, D., & Futter, C. (1986). Assignment of thematic roles to nouns in sentence comprehension by an agrammatic patient. *Brain and Language,**27*(1), 117–134. 10.1016/0093-934X(86)90008-8 3947937 10.1016/0093-934x(86)90008-8

[CR17] Caplan, D., Hildebrandt, N., & Makris, N. (1996). Location of lesions in stroke patients with deficits in syntactic processing in sentence comprehension. *Brain,**119*(3), 933–949. 10.1093/brain/119.3.933 8673503 10.1093/brain/119.3.933

[CR18] Caplan, D., Alpert, N., & Waters, G. (1998). Effects of syntactic structure and propositional number on patterns of regional cerebral blood flow. *Journal of Cognitive Neuroscience,**10*(4), 541–552. 10.1162/089892998562843 9712683 10.1162/089892998562843

[CR19] Caplan, D., Chen, E., & Waters, G. (2008). Task-dependent and task-independent neurovascular responses to syntactic processing. *Cortex,**44*(3), 257–275. 10.1016/j.cortex.2006.06.005 18387556 10.1016/j.cortex.2006.06.005 PMC2427191

[CR20] Caramazza, A., & Zurif, E. B. (1976). Dissociation of algorithmic and heuristic processes in language comprehension: Evidence from aphasia. *Brain and Language,**3*(4), 572–582. 10.1016/0093-934X(76)90048-1 974731 10.1016/0093-934x(76)90048-1

[CR21] Caramazza, A., & Miceli, G. (1991). Selective impairment of thematic role assignment in sentence processing. *Brain and Language,**41*(3), 402–436. 10.1016/0093-934X(91)90164-V 1933265 10.1016/0093-934x(91)90164-v

[CR22] Caramazza, A., Capasso, R., Capitani, E., & Miceli, G. (2005). Patterns of comprehension performance in agrammatic Broca’s aphasia: A test of the trace deletion hypothesis. *Brain and Language,**94*(1), 43–53. 10.1016/j.bandl.2004.11.006 15896382 10.1016/j.bandl.2004.11.006

[CR23] Carreiras, M., Pattamadilok, C., Meseguer, E., Barber, H., & Devlin, J. T. (2012). Broca’s area plays a causal role in morphosyntactic processing. *Neuropsychologia,**50*(5), 816–820. 10.1016/j.neuropsychologia.2012.01.016 22285905 10.1016/j.neuropsychologia.2012.01.016

[CR24] Chang, E. F., Kurteff, G., & Wilson, S. M. (2018). Selective interference with syntactic encoding during sentence production by direct electrocortical stimulation of the inferior frontal gyrus. *Journal of Cognitive Neuroscience,**30*(3), 411–420. 10.1162/jocn_a_01215 29211650 10.1162/jocn_a_01215 PMC5819756

[CR25] Charles, D., Olm, C., Powers, J., Ash, S., Irwin, D. J., McMillan, C. T., Rascovsky, K., & Grossman, M. (2014). Grammatical comprehension deficits in non-fluent/agrammatic primary progressive aphasia. *Journal of Neurology, Neurosurgery & Psychiatry,**85*(3), 249–256. 10.1136/jnnp-2013-305749 24039027 10.1136/jnnp-2013-305749 PMC3925677

[CR26] Cheung, M.W.-L. (2019). A guide to conducting a meta-analysis with non-independent effect sizes. *Neuropsychology Review,**29*(4), 387–396. 10.1007/s11065-019-09415-6 31446547 10.1007/s11065-019-09415-6 PMC6892772

[CR27] Cho-Reyes, S., & Thompson, C. K. (2012). Verb and sentence production and comprehension in aphasia: Northwestern Assessment of Verbs and Sentences (NAVS). *Aphasiology,**26*(10), 1250–1277. 10.1080/02687038.2012.693584 26379358 10.1080/02687038.2012.693584 PMC4569132

[CR28] Dapretto, M., & Bookheimer, S. Y. (1999). Form and content: Dissociating syntax and semantics in sentence comprehension. *Neuron,**24*(2), 427–432. 10.1016/S0896-6273(00)80855-7 10571235 10.1016/s0896-6273(00)80855-7

[CR29] DeLeon, J., Gesierich, B., Besbris, M., Ogar, J., Henry, M. L., Miller, B. L., Gorno-Tempini, M. L., & Wilson, S. M. (2012). Elicitation of specific syntactic structures in primary progressive aphasia. *Brain and Language,**123*(3), 183–190. 10.1016/j.bandl.2012.09.004 23046707 10.1016/j.bandl.2012.09.004 PMC3502680

[CR30] DeMarco, A. T., & Turkeltaub, P. E. (2018). A multivariate lesion symptom mapping toolbox and examination of lesion-volume biases and correction methods in *lesion-sym*pt*om* mapping. *Human Brain Mapping, 39*(11). 10.1002/hbm.24289 10.1002/hbm.24289 PMC6647024 29972618

[CR31] Démonet, J. F., Thierry, G., & Cardebat, D. (2005). Renewal of the neurophysiology of language: Functional neuroimaging. *Physiological Reviews,**85*(1), 49–95. 10.1152/physrev.00049.2003 15618478 10.1152/physrev.00049.2003

[CR32] den Ouden, D. B., Malyutina, S., Basilakos, A., Bonilha, L., Gleichgerrcht, E., Yourganov, G., Hillis, A. E., Hickok, G., Rorden, C., & Fridriksson, J. (2019). Cortical and structural-connectivity damage correlated with impaired syntactic processing in aphasia. *Human Brain Mapping,**40*(7), 2153–2173. 10.1002/hbm.24514 30666767 10.1002/hbm.24514 PMC6445708

[CR33] Dick, F., Bates, E., Wulfeck, B., Utman, J. A., Dronkers, N., & Gernsbacher, M. A. (2001). Language deficits, localization, and grammar: Evidence for a distributive model of language breakdown in aphasic patients and neurologically intact individuals. *Psychological Review,**108*(4), 759. 10.1037/0033-295X.108.4.759 11699116 10.1037/0033-295x.108.4.759 PMC4301444

[CR34] Ditges, R., Barbieri, E., Thompson, C. K., Weintraub, S., Weiller, C., Mesulam, M. M., Kümmerer, D., Schröter, N., & Musso, M. (2021). German language adaptation of the NAVS (NAVS-G) and of the NAT (NAT-G): Testing grammar in aphasia. *Brain Sciences,**11*(4), 474. 10.3390/brainsci11040474 33918022 10.3390/brainsci11040474 PMC8069474

[CR35] Dronkers, N. F., Wilkins, D. P., Van Valin Jr, R. D., Redfern, B. B., & Jaeger, J. J. (2004). Lesion analysis of the brain areas involved in language comprehension. *Cognition,**92*(1–2), 145–177. 10.1016/j.cognition.2003.11.002 15037129 10.1016/j.cognition.2003.11.002

[CR36] Eickhoff, S. B., Nichols, T. E., Laird, A. R., Hoffstaedter, F., Amunts, K., Fox, P. T., Bzdok, D., & Eickhoff, C. R. (2016). Behavior, sensitivity, and power of activation likelihood estimation characterized by massive empirical simulation. *NeuroImage,**137*, 70–85. 10.1016/j.neuroimage.2016.04.072 27179606 10.1016/j.neuroimage.2016.04.072 PMC4981641

[CR37] Faroqi-Shah, Y., Kling, T., Solomon, J., Liu, S., Park, G., & Braun, A. (2014). Lesion analysis of language production deficits in aphasia. *Aphasiology, 28*(3). 10.1080/02687038.2013.853023

[CR38] Fernandino, L., Conant, L. L., Binder, J. R., Blindauer, K., Hiner, B., Spangler, K., & Desai, R. H. (2013). Where is the action? Action sentence processing in Parkinson’s disease. *Neuropsychologia,**51*(8), 1510–1517. 10.1016/j.neuropsychologia.2013.04.008 23624313 10.1016/j.neuropsychologia.2013.04.008 PMC3731159

[CR39] Ferreira, F. (2003). The misinterpretation of noncanonical sentences. *Cognitive Psychology,**47*(2), 164–203. 10.1016/S0010-0285(03)00005-7 12948517 10.1016/s0010-0285(03)00005-7

[CR40] Finocchiaro, C., Capasso, R., Cattaneo, L., Zuanazzi, A., & Miceli, G. (2015). Thematic role assignment in the posterior parietal cortex: A TMS study. *Neuropsychologia,**77*, 223–232. 10.1016/j.neuropsychologia.2015.08.025 26318240 10.1016/j.neuropsychologia.2015.08.025

[CR41] Friederici, A. D., Rüschemeyer, S. A., Hahne, A., & Fiebach, C. J. (2003). The role of left inferior frontal and superior temporal cortex in sentence comprehension: Localizing syntactic and semantic processes. *Cerebral Cortex,**13*(2), 170–177. 10.1093/cercor/13.2.170 12507948 10.1093/cercor/13.2.170

[CR42] Fyndanis, V., Varlokosta, S., & Tsapkini, K. (2013). (Morpho) syntactic comprehension in agrammatic aphasia: Evidence from Greek. *Aphasiology,**27*(4), 398–419. 10.1080/02687038.2013.770817

[CR43] Giglio, L., Ostarek, M., Weber, K., & Hagoort, P. (2022). Commonalities and asymmetries in the neurobiological infrastructure for language production and comprehension. *Cerebral Cortex, 32*(7), 1405–1418. 10.1093/cercor/bhab287 10.1093/cercor/bhab287 PMC8971077 34491301

[CR44] Grimes, N. (2005). *Walt Disney’s Cinderella*. Random House.

[CR45] Goodglass, H., Gleason, J. B., Bernholtz, N. A., & Hyde, M. R. (1972). Some linguistic structures in the speech of a Broca’s aphasic. *Cortex 8*(2), 191–212. 10.1016/S0010-9452(72)80018-2 10.1016/s0010-9452(72)80018-2 5043793

[CR46] Goodglass, H., & Kaplan, E. (1983). *The assessment of aphasia and related disorders*. Lea & Febiger.

[CR47] Grodzinsky, Y. (1986). Language deficits and the theory of syntax. *Brain and Language,**27*(1), 135–159. 10.1016/0093-934X(86)90009-X 3947938 10.1016/0093-934x(86)90009-x

[CR48] Grodzinsky, Y. (2000). The neurology of syntax: Language use without Broca’s area. *Behavioral and Brain Sciences,**23*(1), 1–21. 10.1017/S0140525X00002399 11303337 10.1017/s0140525x00002399

[CR49] Grossman, M., D’Esposito, M., Hughes, E., Onishi, K., Biassou, N., White-Devine, T., & Robinson, K. M. (1996). Language comprehension profiles in Alzheimer’s disease, multi-infarct dementia, and frontotemporal degeneration. *Neurology,**47*(1), 183–189. 10.1212/WNL.47.1.183 8710075 10.1212/wnl.47.1.183

[CR50] Grossman, M., Powers, J., Ash, S., McMillan, C., Burkholder, L., Irwin, D., & Trojanowski, J. Q. (2013). Disruption of large-scale neural networks in non-fluent/agrammatic variant primary progressive aphasia associated with frontotemporal degeneration pathology. *Brain and Language,**127*(2), 106–120. 10.1016/j.bandl.2012.10.005 23218686 10.1016/j.bandl.2012.10.005 PMC3610841

[CR51] Hagiwara, H., & Caplan, D. (1990). Syntactic comprehension in Japanese aphasics: Effects of category and thematic role order. *Brain and Language,**38*(1), 159–170. 10.1016/0093-934X(90)90107-R 1689197 10.1016/0093-934x(90)90107-r

[CR52] Heilman, K. M., & Scholes, R. J. (1976). The nature of comprehension errors in Broca’s, conduction and Wernicke’s aphasics. *Cortex,**12*(3), 258–265. 10.1016/S0010-9452(76)80007-X 1000994 10.1016/s0010-9452(76)80007-x

[CR53] Henseler, I., Regenbrecht, F., & Obrig, H. (2014). Lesion correlates of patholinguistic profiles in chronic aphasia: Comparisons of syndrome-, modality-and symptom-level assessment. *Brain,**137*(3), 918–930. 10.1093/brain/awt374 24525451 10.1093/brain/awt374

[CR54] Henry, M. (2009). *Progressive aphasia: Patterns of language behavior and regional cortical atrophy* [The University of Arizona]. Retrieved November 2023 from https://repository.arizona.edu/handle/10150/196034. Accessed Nov 2022

[CR55] Hu, J., Small, H., Kean, H., Takahashi, A., Zekelman, L., Kleinman, D., Ryan, E., Nieto- Castañón, A., Ferreira, V., & Fedorenko, E. (2023). Precision fMRI reveals that the language-selective network supports both phrase-structure building and lexical access during language production. *Cerebral Cortex, 33*(8), 4384–4404. 10.1093/cercor/bhac350 10.1093/cercor/bhac350 PMC10110436 36130104

[CR56] Huber, W., Poeck, K., & Willmes, K. (1984). The Aachen aphasia test. *Advances in Neurology,**42*, 291–303. 6209953

[CR57] Kaan, E., & Swaab, T. Y. (2002). The brain circuitry of syntactic comprehension. *Trends in Cognitive Sciences,**6*(8), 350–356. 10.1016/S1364-6613(02)01947-2 12140086 10.1016/s1364-6613(02)01947-2

[CR58] Kamminga, J., Leslie, F. V. C., Hsieh, S., Caga, J., Mioshi, E., Hornberger, M., Ballard, K. J., Kiernan, M. C., Hodges, J. R., & Burrell, J. R. (2016). Syntactic comprehension deficits across the FTD-ALS continuum. *Neurobiology of Aging,**41*, 11–18. 10.1016/j.neurobiolaging.2016.02.002 27103514 10.1016/j.neurobiolaging.2016.02.002

[CR59] Kertesz, A. (1982). *Western aphasia battery*. Grune and Stratton.

[CR60] Kertesz, A. (2007). *Western aphasia battery - Revised*. San Antonio, TX: Pearson.

[CR61] Kim, M. J., Jeon, H. A., & Lee, K. M. (2010). Impairments of syntactic comprehension in Korean and the location of ischemic stroke lesions: A voxel-based lesion-symptom mapping study. *Behavioural Neurology,**22*(1–2), 3–10. 10.3233/BEN-2009-0254 20543453 10.3233/BEN-2009-0254 PMC5434407

[CR62] Kinno, R., Muragaki, Y., Hori, T., Maruyama, T., Kawamura, M., & Sakai, K. L. (2009). Agrammatic comprehension caused by a glioma in the left frontal cortex. *Brain and Language,**110*(2), 71–80. 10.1016/j.bandl.2009.05.001 19573900 10.1016/j.bandl.2009.05.001

[CR63] Kinno, R., Ohta, S., Muragaki, Y., Maruyama, T., & Sakai, K. L. (2014). Differential reorganization of three syntax-related networks induced by a left frontal glioma. *Brain,**137*(4), 1193–1212. 10.1093/brain/awu013 24519977 10.1093/brain/awu013

[CR64] Kristinsson, S., Thors, H., Yourganov, G., Magnusdottir, S., Hjaltason, H., Stark, B. C., Basilakos, A., den Ouden, D.-B., Bonilha, L., Rorden, C., Hickok, G., Hillis, A., & Fridriksson, J. (2020). Brain damage associated with impaired sentence processing in acute aphasia. *Journal of Cognitive Neuroscience*, *32*(2). 10.1162/jocn_a_01478 10.1162/jocn_a_01478 PMC7132331 31596169

[CR65] LaCroix, A. N., Blumenstein, N., Tully, M., Baxter, L. C., & Rogalsky, C. (2020). Effects of prosody on the cognitive and neural resources supporting sentence comprehension: A behavioral and lesion-symptom mapping study. *Brain and Language,**203*, 104756. 10.1016/j.bandl.2020.104756 32032865 10.1016/j.bandl.2020.104756 PMC7064294

[CR66] Lenneberg, E. H. (1973). The neurology of language. *Daedalus*, *102*(3), 115–133. Retrieved January 2023 from https://www.jstor.org/stable/20024149. Accessed Jan 2023

[CR67] Locke, S., Caplan, D., & Kellar, L. A., (1973). *A study in neurolinguistics*. Springfield, IL: Charles C. Thomas.

[CR68] Love, T., & Oster, E. (2002). On the categorization of aphasic typologies: The SOAP (a test of syntactic complexity). *Journal of Psycholinguistic Research,**31*, 503–529. 12528429 10.1023/a:1021208903394

[CR69] Lukic, S., Barbieri, E., Wang, X., Caplan, D., Kiran, S., Rapp, B., Parrish, T. B., & Thompson, C. K. (2017). Right hemisphere grey matter volume and language functions in stroke aphasia. *Neural Plasticity, 2017*. 10.1155/2017/5601509 10.1155/2017/5601509 PMC5441122 28573050

[CR70] Lukic, S., Thompson, C. K., Barbieri, E., Chiappetta, B., Bonakdarpour, B., Kiran, S., Rapp, B., Parrish, T. B., & Caplan, D. (2020). Common and distinct neural substrates of sentence production and comprehension. *NeuroImage,**224*, 117374. 10.1016/j.neuroimage.2020.117374 32949711 10.1016/j.neuroimage.2020.117374 PMC10134242

[CR71] Mack, J. E., Meltzer-Asscher, A., Barbieri, E., & Thompson, C. K. (2013). Neural correlates of processing passive sentences. *Brain Sciences,**3*(3), 1198–1214. 10.3390/brainsci3031198 24961525 10.3390/brainsci3031198 PMC4061884

[CR72] Magnusdottir, S. (2005). *Setningafræðiprof (Test of Syntax)*. Landspıtali - University Hospital.

[CR73] Magnusdottir, S., Fillmore, P., Den Ouden, D. B., Hjaltason, H., Rorden, C., Kjartansson, O., Bonilha, L., & Fridriksson, J. (2013). Damage to left anterior temporal cortex predicts impairment of complex syntactic processing: A lesion-symptom mapping study. *Human Brain Mapping,**34*(10), 2715–2723. 10.1002/hbm.22096 22522937 10.1002/hbm.22096 PMC6869931

[CR74] Maher, L. M., Chatterjee, A., Rothi, L. G., & Heilman, K. M. (1995). Agrammatic sentence production: The use of a temporal-spatial strategy. *Brain and Language,**49*(2), 105–124. 10.1006/brln.1995.1023 7648247 10.1006/brln.1995.1023

[CR75] Matchin, W., Basilakos, A., Stark, B. C., den Ouden, D. B., Fridriksson, J., & Hickok, G. (2020). Agrammatism and paragrammatism: A cortical double dissociation revealed by lesion-symptom mapping. *Neurobiology of Language,**1*(2), 208–225. 10.1162/nol_a_00010 34296193 10.1162/nol_a_00010 PMC8293792

[CR76] Matchin, W., Ouden, D.-B., den Hickok, G., Hillis, A. E., Bonilha, L., & Fridriksson, J. (2021). The Wernicke conundrum revisited: Evidence from connectome-based lesion-symptom mapping. *bioRxiv*, 2021–10. 10.1101/2021.10.25.465746 10.1093/brain/awac219 PMC10200287 35727949

[CR77] Matchin, W., Basilakos, A., Den Ouden, D. B., Stark, B. C., Hickok, G., & Fridriksson, J. (2022). Functional differentiation in the language network revealed by lesion-symptom mapping. *NeuroImage,**247*, 118778. 10.1016/j.neuroimage.2021.118778 34896587 10.1016/j.neuroimage.2021.118778 PMC8830186

[CR78] Meltzer, J. A., & Braun, A. R. (2011). An EEG-MEG dissociation between online syntactic comprehension and post hoc reanalysis. *Frontiers in Human Neuroscience,**5*, 10. 10.3389/fnhum.2011.00010 21331355 10.3389/fnhum.2011.00010 PMC3035013

[CR79] Meltzer, J. A., Wagage, S., Ryder, J., Solomon, B., & Braun, A. R. (2013). Adaptive significance of right hemisphere activation in aphasic language comprehension. *Neuropsychologia,**51*(7), 1248–1259. 10.1016/j.neuropsychologia.2013.03.007 23566891 10.1016/j.neuropsychologia.2013.03.007 PMC3821997

[CR80] Meltzer-Asscher, A., Schuchard, J., den Ouden, D. B., & Thompson, C. K. (2013). The neural substrates of complex argument structure representations: Processing “alternating transitivity” verbs. *Language and Cognitive Processes,**28*(8), 1154–1168. 10.1080/01690965.2012.672754 26139954 10.1080/01690965.2012.672754 PMC4485426

[CR81] Menn, L., Obler, L., & Goodglass, H. (1990). *A cross-language study of agrammatism*. Benjamins.

[CR82] Mesulam, M. M., Coventry, C. A., Rader, B. M., Kuang, A., Sridhar, J., Martersteck, A., Zhang, H., Thompson, C. K., Weintraub, S., & Rogalski, E. J. (2021). Modularity and granularity across the language network-a primary progressive aphasia perspective. *Cortex,**141*, 482–496. 10.1016/j.cortex.2021.05.002 34153680 10.1016/j.cortex.2021.05.002 PMC8319115

[CR83] Meyer, A. M., Mack, J. E., & Thompson, C. K. (2012). Tracking passive sentence comprehension in agrammatic aphasia. *Journal of Neurolinguistics,**25*(1), 31–43. 10.1016/j.jneuroling.2011.08.001 22043134 10.1016/j.jneuroling.2011.08.001 PMC3203204

[CR84] Mayer, M. F. (1969). *Where are you?* Penguin Books.

[CR85] Miceli, G., Mazzucchi, A., Menn, L., & Goodglass, H. (1983). Contrasting cases of Italian agrammatic aphasia without comprehension disorder. *Brain and Language,**19*(1), 65–97. 10.1016/0093-934X(83)90056-1 6860936 10.1016/0093-934x(83)90056-1

[CR86] Miceli, G., Silveri, M. C., Romani, C., & Caramazza, A. (1989). Variation in the pattern of omissions and substitutions of grammatical morphemes in the spontaneous speech of so-called agrammatic patients. *Brain and Language,**36*(3), 447–492. 10.1016/0093-934X(89)90079-5 2706449 10.1016/0093-934x(89)90079-5

[CR87] Miozzo, M., Fischer-Baum, S., & Postman, J. (2008). Knowing where but not what: Impaired thematic roles and spatial language. *Cognitive Neuropsychology,**25*(6), 853–873. 10.1080/02643290802365151 18792829 10.1080/02643290802365151

[CR88] Mirman, D., Kraft, A. E., Harvey, D. Y., Brecher, A. R., & Schwartz, M. F. (2019). Mapping articulatory and grammatical subcomponents of fluency deficits in post-stroke aphasia. *Cognitive, Affective, & Behavioral Neuroscience,**19*(5), 1286–1298. 10.3758/s13415-019-00729-9 10.3758/s13415-019-00729-9 PMC6786948 31240565

[CR89] Moore, M. J., Demeyere, N., Rorden, C., & Mattingley, J. B. (2024). Lesion mapping in neuropsychological research: A practical and conceptual guide. *Cortex; a Journal Devoted to the Study of the Nervous System and Behavior,**170*, 38–52. 10.1016/j.cortex.2023.10.001 37940465 10.1016/j.cortex.2023.10.001 PMC11474248

[CR90] Moro, A., Tettamanti, M., Perani, D., Donati, C., Cappa, S. F., & Fazio, F. (2001). Syntax and the brain: Disentangling grammar by selective anomalies. *NeuroImage,**13*(1), 110–118. 10.1006/nimg.2000.0668 11133314 10.1006/nimg.2000.0668

[CR91] Müller, V. I., Cieslik, E. C., Laird, A. R., Fox, P. T., Radua, J., Mataix-Cols, D., Tench, C. R., Yarkoni, T., Nichols, T. E., Turkeltaub, P. E., Wager, T. D., & Eickhoff, S. B. (2018). Ten simple rules for neuroimaging meta-analysis. *Neuroscience & Biobehavioral Reviews,**84*, 151–161. 10.1016/j.neubiorev.2017.11.012 29180258 10.1016/j.neubiorev.2017.11.012 PMC5918306

[CR92] Na, Y., Jung, J., Tench, C. R., Auer, D. P., & Pyun, S. B. (2022). Language systems from lesion-symptom mapping in aphasia: A meta-analysis of voxel-based lesion mapping studies. *NeuroImage: Clinical*, *35*, 103038. 10.1016/j.nicl.2022.103038 10.1016/j.nicl.2022.103038 PMC9112051 35569227

[CR93] Nespoulous, J. L., Dordain, M., Perron, C., Ska, B., Bub, D., Caplan, D., Mehler, J., & Lecours, A. R. (1988). Agrammatism in sentence production without comprehension deficits: Reduced availability of syntactic structures and/or of grammatical morphemes? *A Case Study. Brain and Language,**33*(2), 273–295. 10.1016/0093-934X(88)90069-7 3359172 10.1016/0093-934x(88)90069-7

[CR94] Newhart, M., Trupe, L. A., Gomez, Y., Cloutman, L., Molitoris, J. J., Davis, C., Leigh, R., Gottesman, R. F., Race, D., & Hillis, A. E. (2012). Asyntactic comprehension, working memory, and acute ischemia in Broca’s area versus angular gyrus. *Cortex,**48*(10), 1288–1297. 10.1016/j.cortex.2011.09.009 22079684 10.1016/j.cortex.2011.09.009 PMC3389171

[CR95] Nilipour, R., & Raghibdoust, S. (2001). Manifestations of aphasia in Persian. *Journal of Neurolinguistics,**14*(2–4), 209–230. 10.1016/S0911-6044(01)00015-X

[CR96] Novick, J. M., Trueswell, J. C., & Thompson-Schill, S. L. (2005). Cognitive control and parsing: Reexamining the role of Broca’s area in sentence comprehension. *Cognitive, Affective, & Behavioral Neuroscience,**5*(3), 263–281. 10.3758/CABN.5.3.263 10.3758/cabn.5.3.263 16396089

[CR97] Ostrin, R. K., & Tyler, L. K. (1995). Dissociations of lexical function: Semantics, syntax, and morphology. *Cognitive Neuropsychology,**12*(4), 345–389.

[CR98] Page, M. J., McKenzie, J. E., Bossuyt, P. M., Boutron, I., Hoffmann, T. C., Mulrow, C. D., Shamseer, L., Tetzlaff, J. M., Akl, E. A., Brennan, S. E., Chou, R., Glanville, J., Grimshaw, J. M., Hróbjartsson, A., Lalu, M. M., Li, T., Loder, E. W., Mayo-Wilson, E., McDonald, S., . . . Moher, D. (2021). The PRISMA 2020 statement: An updated guideline for reporting systematic reviews. *BMJ, 372*, n71. 10.1136/bmj.n71 10.1136/bmj.n71 PMC8005924 33782057

[CR99] Peelle, J. E., Troiani, V., Gee, J., Moore, P., McMillan, C., Vesely, L., & Grossman, M. (2008). Sentence comprehension and voxel-based morphometry in progressive nonfluent aphasia, semantic dementia, and nonaphasic frontotemporal dementia. *Journal of Neurolinguistics, 21*(5), Art. 5. 10.1016/j.jneuroling.2008.01.004 10.1016/j.jneuroling.2008.01.004 PMC2598754 19727332

[CR100] Pettigrew, C., & Hillis, A. E. (2014). Role for memory capacity in sentence comprehension: Evidence from acute stroke. *Aphasiology,**28*(10), 1258–1280. 10.1080/02687038.2014.919436 25221377 10.1080/02687038.2014.919436 PMC4158714

[CR101] Pickles, J. O. (2015). Chapter 1 - Auditory pathways: Anatomy and physiology. In M. J. Aminoff, F. Boller, & D. F. Swaab (Eds.), *Handbook of Clinical Neurology* (Vol. 129, pp. 3–25). Elsevier. 10.1016/B978-0-444-62630-1.00001-9 10.1016/B978-0-444-62630-1.00001-9 25726260

[CR102] Pillay, S. B., Binder, J. R., Humphries, C., Gross, W. L., & Book, D. S. (2017). Lesion localization of speech comprehension deficits in chronic aphasia. *Neurology,**88*(10), 970–975. 10.1212/WNL.0000000000003683 28179469 10.1212/WNL.0000000000003683 PMC5333516

[CR103] Price, C. J. (2012). A review and synthesis of the first 20 years of PET and fMRI studies of heard speech, spoken language and reading. *NeuroImage,**62*(2), 816–847. 10.1016/j.neuroimage.2012.04.062 22584224 10.1016/j.neuroimage.2012.04.062 PMC3398395

[CR104] Riccardi, N., Rorden, C., Fridriksson, J., & Desai, R. H. (2022). Canonical sentence processing and the inferior frontal cortex: Is there a connection? *Neurobiology of Language,**3*(2), 318–344. 10.1162/nol_a_00067 37215558 10.1162/nol_a_00067 PMC10158581

[CR105] Richardson, F. M., Thomas, M. S., & Price, C. J. (2010). Neuronal activation for semantically reversible sentences. *Journal of Cognitive Neuroscience,**22*(6), 1283–1298. 10.1162/jocn.2009.21277 19445603 10.1162/jocn.2009.21277 PMC2860570

[CR106] Rogalski, E., Cobia, D., Harrison, T. M., Wieneke, C., Thompson, C. K., Weintraub, S., & Mesulam, M. M. (2011). Anatomy of language impairments in primary progressive aphasia. *Journal of Neuroscience,**31*(9), 3344–3350. 10.1523/JNEUROSCI.5544-10.2011 21368046 10.1523/JNEUROSCI.5544-10.2011 PMC3112000

[CR107] Rogalsky, C., LaCroix, A. N., Chen, K. H., Anderson, S. W., Damasio, H., Love, T., & Hickok, G. (2018). The neurobiology of agrammatic sentence comprehension: A lesion study. *Journal of Cognitive Neuroscience,**30*(2), 234–325. 10.1162/jocn_a_01200 29064339 10.1162/jocn_a_01200 PMC6434535

[CR108] Saffran, E. M., Schwartz, M. F., & Marin, O. S. M. (1980). The word order problem in agrammatism: II. *Production. Brain and Language,**10*(2), 263–280. 10.1016/0093-934X(80)90056-5 10.1016/0093-934x(80)90056-5 7407547

[CR109] Saffran, E. M., Schwartz, M. F., Linebarger, M. C., Martin, N., & Bochetto, P. (1988). *The Philadelphia comprehension battery*. Unpublished test battery.

[CR110] Saffran, E. M., Berndt, R. S., & Schwartz, M. F. (1989). The quantitative analysis of agrammatic production: Procedure and data. *Brain and Language,**37*(3), 440–479. 10.1016/0093-934X(89)90030-8 2804622 10.1016/0093-934x(89)90030-8

[CR111] Sapolsky, D., Bakkour, A., Negreira, A., Nalipinski, P., Weintraub, S., Mesulam, M.-M., Caplan, D., & Dickerson, B. C. (2010). Cortical neuroanatomic correlates of symptom severity in primary progressive aphasia. *Neurology,**75*(4), 358–366. 10.1212/WNL.0b013e3181ea15e8 20660866 10.1212/WNL.0b013e3181ea15e8 PMC2918888

[CR112] Schwartz, M. F., Saffran, E. M., & Marin, O. S. (1980). The word order problem in agrammatism: I. *Comprehension. Brain and Language,**10*(2), 249–262. 10.1016/0093-934X(80)90055-3 7407546 10.1016/0093-934x(80)90055-3

[CR113] Sheppard, S. M., Meier, E. L., Kim, K. T., Breining, B. L., Keator, L. M., Tang, B., Caffo, B. S., & Hillis, A. E. (2022). Neural correlates of syntactic comprehension: A longitudinal study. *Brain and Language,**225*, 105068. 10.1016/j.bandl.2021.105068 34979477 10.1016/j.bandl.2021.105068 PMC9232253

[CR114] Shiani, A., Joghataei, M. T., Ashayeri, H., Kamali, M., Razavi, M. R., Yadegari, F. (2019). Comprehension of complex sentences in the Persian-speaking patients with aphasia. *Basic and Clinical Neuroscience, 10*(3), 199. 10.32598/bcn.9.10.185 10.32598/bcn.9.10.185 PMC6712637 31462975

[CR115] Slobin, D. I. (1991). Aphasia in Turkish: Speech production in Broca’s and Wernicke’s patients. *Brain and Language,**41*(2), 149–164. 10.1016/0093-934X(91)90150-Y 1933256 10.1016/0093-934x(91)90150-y

[CR116] Stefaniak, J. D., Alyahya, R. S., & Ralph, M. A. L. (2021). Language networks in aphasia and health: A 1000 participant activation likelihood estimation meta-analysis. *NeuroImage,**233*, 117960. 10.1016/j.neuroimage.2021.117960 33744459 10.1016/j.neuroimage.2021.117960

[CR117] Stromswold, K., Caplan, D., Alpert, N., & Rauch, S. (1996). Localization of syntactic comprehension by positron emission tomography. *Brain and Language,**52*(3), 452–473. 10.1006/brln.1996.0024 8653390 10.1006/brln.1996.0024

[CR118] Thompson, C. K., & Mack, J. E. (2014). Grammatical Impairments in PPA. *Aphasiology,**28*(8–9), 1018–1037. 10.1080/02687038.2014.912744 25642014 10.1080/02687038.2014.912744 PMC4306464

[CR119] Thompson, C. K., Fix, S., & Gitelman, D. (2002). Selective impairment of morphosyntactic production in a neurological patient. *Journal of Neurolinguistics,**15*(3–5), 189–207. 10.1016/S0911-6044(01)00038-0 21274411 10.1016/S0911-6044(01)00038-0 PMC3026292

[CR120] Thompson, C. K., Weintraub, S., & Mesulam, M. (2012). *Northwestern anagram test (NAT) Northwestern University*. Evanston, IL, USA.

[CR121] Thothathiri, M., Kimberg, D. Y., & Schwartz, M. F. (2012). The neural basis of reversible sentence comprehension: Evidence from voxel-based lesion symptom mapping in aphasia. *Journal of Cognitive Neuroscience,**24*(1), 212–222. 10.1162/jocn_a_00118 21861679 10.1162/jocn_a_00118 PMC3389786

[CR122] Tissot, R., Mounin, G., Lhermitte. F. (1973). *L’agrammatisme*. Bruxelles, Charles Dessart.

[CR123] Tyler, L. K., Marslen-Wilson, W. D., Randall, B., Wright, P., Devereux, B. J., Zhuang, J., Papoutsi, M., & Stamatakis, E. A. (2011). Left inferior frontal cortex and syntax: Function, structure and behaviour in patients with left hemisphere damage. *Brain,**134*(2), 415–431. 10.1093/brain/awq369 21278407 10.1093/brain/awq369 PMC3030769

[CR124] Tzourio-Mazoyer, N., Landeau, B., Papathanassiou, D., Crivello, F., Etard, O., Delcroix, N., & Mazoyer., B., Joliot, M. (2002). Automated anatomical labeling of activations in SPM using a macroscopic anatomical parcellation of the MNI MRI single-subject brain. *NeuroImage,**15*(1), 273–289. 10.1006/nimg.2001.0978 11771995 10.1006/nimg.2001.0978

[CR125] Vercesi, L., Sabnis, P., Finocchiaro, C., Cattaneo, L., Tonolli, E., & Miceli, G. (2020). The role of the l-IPS in the comprehension of reversible and irreversible sentences: An rTMS study. *Brain Structure and Function,**225*, 2403–2414. 10.1007/s00429-020-02130-6 32844277 10.1007/s00429-020-02130-6 PMC7544754

[CR126] Vigneau, M., Beaucousin, V., Hervé, P. Y., Jobard, G., Petit, L., Crivello, F., Mellet, E., Zago, L., Mazoyer, B., & Tzourio-Mazoyer, N. (2011). What is right-hemisphere contribution to phonological, lexico-semantic, and sentence processing?: Insights from a meta-analysis. *NeuroImage,**54*(1), 577–593. 10.1016/j.neuroimage.2010.07.036 20656040 10.1016/j.neuroimage.2010.07.036

[CR127] Walenski, M., Europa, E., Caplan, D., & Thompson, C. K. (2019). Neural networks for sentence comprehension and production: An ALE-based meta-analysis of neuroimaging studies. *Human Brain Mapping,**40*(8), 2275–2304. 10.1002/hbm.24523 30689268 10.1002/hbm.24523 PMC6597252

[CR128] Wassenaar, M., & Hagoort, P. (2007). Thematic role assignment in patients with Broca’s aphasia: Sentence–picture matching electrified. *Neuropsychologia,**45*(4), 716–740. 10.1016/j.neuropsychologia.2006.08.016 17005212 10.1016/j.neuropsychologia.2006.08.016

[CR129] Weintraub, S., Mesulam, M.-M., Wieneke, C., Rademaker, A., Rogalski, E. J., & Thompson, C. K. (2010). The northwestern anagram test: Measuring sentence production in primary progressive aphasia. *American Journal of Alzheimer’s Disease and Other Dementias,**24*, 408–416. 10.1177/1533317509343104 10.1177/1533317509343104 PMC2836907 19700669

[CR130] Westbury, C. (2015). *ALFAB: The Alberta language function assessment battery [online]*. Retrieved January 2023 from https://www.psych.ualberta.ca/~westburylab/downloads/alfab.download. Accessed Jan 2023

[CR131] Wilson, S. M., Henry, M. L., Besbris, M., Ogar, J. M., Dronkers, N. F., Jarrold, W., Miller, B. L., & Gorno-Tempini, M. L. (2010a). Connected speech production in three variants of primary progressive aphasia. *Brain,**133*(7), 2069–2088. 10.1093/brain/awq129 20542982 10.1093/brain/awq129 PMC2892940

[CR132] Wilson, S. M., Dronkers, N. F., Ogar, J. M., Jang, J., Growdon, M. E., Agosta, F., Henry, M. L., Miller, B. L., & Gorno-Tempini, M. L. (2010b). Neural correlates of syntactic processing in the nonfluent variant of primary progressive aphasia. *Journal of Neuroscience,**30*(50), 16845–16854. 10.1523/JNEUROSCI.2547-10.2010 21159955 10.1523/JNEUROSCI.2547-10.2010 PMC3024013

[CR133] Wilson, S. M., Galantucci, S., Tartaglia, M. C., Rising, K., Patterson, D. K., Henry, M. L., Ogar, J. M., DeLeon, J., Miller, B. L., & Gorno-Tempini, M. L. (2011). Syntactic Processing depends on dorsal language tracts. *Neuron,**72*(2), 397–403. 10.1016/j.neuron.2011.09.014 22017996 10.1016/j.neuron.2011.09.014 PMC3201770

[CR134] Wilson, S. M., DeMarco, A. T., Henry, M. L., Gesierich, B., Babiak, M., Miller, B. L., & Gorno-Tempini, M. L. (2016). Variable disruption of a syntactic processing network in primary progressive aphasia. *Brain,**139*(11), 2994–3006. 10.1093/brain/aww218 27554388 10.1093/brain/aww218 PMC5091045

[CR135] Wu, D. H., Waller, S., & Chatterjee, A. (2007). The functional neuroanatomy of thematic role and locative relational knowledge. *Journal of Cognitive Neuroscience,**19*(9), 1542–1555. 10.1162/jocn.2007.19.9.1542 17714015 10.1162/jocn.2007.19.9.1542

[CR136] Zurif, E., Swinney, D., Prather, P., Solomon, J., & Bushell, C. (1993). An on-line analysis of syntactic processing in Broca’s and Wernicke’s aphasia. *Brain and Language,**45*(3), 448–464. 10.1006/brln.1993.1054 8269334 10.1006/brln.1993.1054

