# Supplementary material for: The Neurofunctional Correlates of Morphosyntactic and Thematic Impairments in Aphasia: A Systematic Review and Meta-analysis
Source: Neuropsychol Rev. 2024 Aug 31;35(3):483–516. doi: 10.1007/s11065-024-09648-0 (PMC12602653; doi:10.1007/s11065-024-09648-0)
Supplement: Supplementary file 1 — Supplementary file1 (DOCX 9 KB) [file 11065_2024_9648_MOESM1_ESM.docx]

**#Title:**

**THE NEUROFUNCTIONAL CORRELATES OF MORPHOSYNTACTIC AND THEMATIC IMPAIRMENTS IN APHASIA: A SYSTEMATIC REVIEW AND META-ANALYSIS**

**Supplementary Text**

(Please refer to Supplementary Table 1 for the contents described here).

**Supplementary sensitivity meta-analyses**

Some of the selected studies for our systematic literature review and meta-analysis used partially overlapping samples (see Discussion Section in the main text).

To control for possible biases in counting studies with partially overlapping samples, we carefully inspected the result output produced by the GingerALE software. This output provides information on the contribution of every individual study to the significant meta-analytic clusters, thus allowing to estimate the likely impact of excluding one or more studies form the meta-analysis. We sought confirmation for these estimates through a sensitivity analysis approach, in which we repeated every meta-analysis after the exclusion of combinations of studies yielding sample overlaps, including the least favourable combination as projected by GingerALE (Supplementary Table 1).

**Structure of Supplementary Table 1 file**

*Sheet “ListOfSampleOverlaps"*

This file sheet summarizes information regarding sample overlap among studies included in our meta-analyses, along with the corresponding number of overlapping participants (when reported in the original papers).

In column E, we also report the set of meta-analyses of our study for which each sample overlap is relevant.

*Sheets "Meta-analysis1", "Meta-analysis2", "Meta-analysis3"*

These sheets provide three different sets of tables each.

The table on the left reports the GingerALE cluster table for the meta-analysis of interest. This table, which is part of the standard result output of the GingerALE software (version 3.0.2; https://brainmap.org/ale/), provides information on the contribution of every individual study to the significant meta-analytic clusters. The quantitative contribution of each study to each cluster is represented by the number of contributed foci.

The set of tables in the middle reports the list of all possible options to remove overlapping samples from the meta-analysis of interest.

The set of tables on the right summarizes the projected impact of each of the options listed in the middle on each of the significant meta-analysis clusters, based on the GingerALE cluster table. Clusters affected by all removed studies are highlighted in orange. Clusters that are not affected by any of the removed studies are highlighted in green.

For each meta-analysis, we opted to carry out a set of two sensitivity analyses: one implementing the theoretically least favourable option, and the other one the most favourable option, based on the projections summarized in the set of tables on the right side. These two options are highlighted in cyan.

*Sheets "Meta-analysis1_Option5", "Meta-analysis1_Option6", "Meta-analysis2_Option1", "Meta-analysis2_Option2", "Meta-analysis3_Option2", "Meta-analysis3_Option3"*

These sheets report the result of the sensitivity analyses we carried out, in comparison to the results of the corresponding meta-analyses reported in Table 6 of the main text.

On the top side of each sheet, we report for convenience of the reader the same information as in the previous sheets (i.e., the selected option to remove overlapping samples, and the projected impact of this option on the significant clusters).

On the bottom left, we report the table with the results of the meta-analysis of interest (this information is the same as reported in Table 6 of the manuscript).

On the bottom right, we report the results of the target sensitivity analysis.

Brain areas found in the meta-analysis but not in the sensitivity analysis (or vice-versa), are highlighted in grey.
